# Interventions for health equity with a One Health focus: a review of reviews

**DOI:** 10.3389/fpubh.2026.1736987

**Published:** 2026-04-13

**Authors:** Hélène Delisle, Angélique Ingabire, Lene Søvold, Bilkis Vissandjee

**Affiliations:** 1 Département de Nutrition, Faculté de Médecine, Université de Montréal, Montréal, QC, Canada; 2 École de Santé Publique, Université de Montréal, Montréal, QC, Canada; 3 Independent Researcher, Oslo, Norway; 4 Faculté des Sciences Infirmières et Centre de Recherche en Santé Publique, Institut Universitaire SHERPA, Université de Montréal, Montréal, QC, Canada

**Keywords:** health equity, health equity actions, One Health, planetary health, sustainable health

## Abstract

**Introduction:**

The One Sustainable Health for All (OSH) Forum was launched in 2021 to promote a transdisciplinary “One Health/Planetary Health” approach in line with the 2030 Sustainable Development Goals. The “One Health” approach is a holistic and system-based approach that recognizes the interconnection between health of humans, animals and ecosystems. The OSH Forum leads thematic international working groups (IWGs), and the IWG on health equity undertook a scoping review as part of its mandate. This scoping review of reviews focused on actions to achieve health equity in the realm of One Health. The aim was to describe the types of health equity actions, to identify knowledge gaps and to recommend approaches integrating health equity and One Health.

**Methods:**

The literature search only included peer-reviewed action-focused papers. The WHO building blocks were adapted to categorize the lines of action into five key areas.

**Results:**

We analyzed 62 reviews out of 295 action-focused papers. Predominant actions were in the area of service delivery (26/62 reviews). Health equity was addressed through governance in 13 reviews, information/evidence data in 7, technologies in 11, and human resources in 5. Refugees, immigrants, and racial/ethnic minorities were the main targeted communities. The connection of health equity and One Health was not directly addressed except in two reviews. Nearly all the reviews were from high-income countries. Few studies assessed the impact of the interventions on health equity. Recurrent themes across the reviews were: the importance of addressing the social determinants of health; the need for disaggregated data; the critical role of human resources and community engagement; and the need to analyze power imbalances.

**Conclusion:**

The review highlighted a dire need for studies on the impact of interventions on health equity. Given the limited connections made between health equity and One Health, using a health equity lens to assess One Health initiatives, and vice versa, appears warranted.

## 1 Introduction

“Those who make an enemy of the earth make an enemy of themselves” (1). However, harsh it may sound, this best describes the current reality. The human population continues to destroy the planet, our only home. We are now facing extreme climatic events, pandemic threats, rising conflicts throughout the world, and soaring social inequities. On this backdrop, the One Sustainable Health (OSH) for All Forum was launched in 2021 to promote a transdisciplinary, multidisciplinary, and interdisciplinary “One Health/Planetary Health” approach in line with the 2030 Sustainable Development Goals (SDGs). The “*One Health*” (OH) approach is a holistic and system-based approach that recognizes the interconnection between the health of humans, animals, and ecosystems (2). The approach mobilizes multiple sectors, disciplines, and communities at varying levels of society to work together to foster wellbeing and tackle threats to health and ecosystems. Collaboration is a key principle of the OH, and equity must be prioritized in OH implementation to ensure the active participation of vulnerable groups (3). By reconnecting human beings, animal populations, and the environment, the One Health approach brings the possibility for the human population to live in better harmony with nature and other species. “One *Sustainable* Health” emphasizes the need to address the long-term impacts of policies and practices across human, animal, and planetary health. Central to the concept of “One Sustainable Health *for all*” is health equity, which implies fair access of all human beings (and animals) to quality health-related services and the health outcomes achieved. Inherent in this concept is “Universal Health Coverage” (UHC), which claims that all individuals and communities should receive the health services they need without suffering debilitating financial hardship (4). UHC commits to ensuring the highest attainable level of health for all. It includes the full spectrum of essential, quality health services, encompassing health promotion and prevention, treatment, rehabilitation, and palliative care across the life course. The OSH Forum, however, takes this idea further by including nature and natural systems, which includes the human population as well as the rest of the animal kingdom and natural systems, that is, *Planetary health* (5).

Within this context, the OSH Forum launched nine working groups for activities to operationalize these principles.^1^ One of the activities is a scoping review of initiatives to improve health equity with a One Health focus. Health equity and One Health initiatives can enhance the ability to meet the health-related SDGs, especially in reducing health disparities and addressing global health threats (6, 7). Almost all SDGs are connected to health, and some in a direct manner: SDG 2 Zero hunger; SDG 3 Good health and wellbeing; SDG 5 Gender equality; SDG 6 Clean water and sanitation; SDG 10 Reduced inequalities; SDG 11 Sustainable cities and communities; and SDG 13 Climate action. The point here is that all the SDGs are mainly human-centered and there is a need to think and act beyond that.

Health equity generally refers to the absence of unfair and avoidable differences in health among population groups defined socially, economically, demographically, and/or geographically (8).

Conversely, WHO (9) defines health inequities as differences in health status or in the distribution of health resources between different population groups, arising from the social conditions in which people are born, grow, live, work, and age. The term “global health equity” can also be used to describe equitable health as a key outcome of global health activities (10). Health equity covers not only health but also its determinants (11). The components or dimensions of health equity are the access to health services, the quality of those services and the health outcomes.

Increased focus on health equity is important, particularly now with rising inequities, given that there are population groups who systematically experience lower access to health services, lower health status, limited wellbeing outcomes, and higher exposure to risks and stressors in many countries all over the world (12). For instance, persons living with disabilities−16% of the world population -, experience a life expectancy shortened by 10–20 years, as they are more exposed (13). The recent COVID-19 pandemic dramatically showed that mortality and morbidity followed a social gradient (14). Profound inequalities in access to the COVID-19 vaccines and the adverse consequences, including in mental health, were also highlighted. It is also important to highlight that COVID-19 provided evidence that without human interference, nature has the capacity to flourish. For example, due to COVID-19, the quality of air and water increased, and landscapes became cleaner.

The purpose of the scoping review of initiatives to improve health equity was to describe the different types of health equity actions, to identify knowledge gaps, and to recommend approaches integrating health equity and One Health.

## 2 Methodology

The initial literature search on health equity actions was conducted in June 2022 and a search update in April 2024. Only peer-reviewed papers published since the year 2000 in English or French were included. We used the following databases: PubMed; Embase (Elsevier); APA PsycInfo; CINAHL; Érudit; Global Health; Social Sciences Abstracts; Sociological Abstracts; and Web of Science (WoS). The final concept plan and the search words are given in Appendix. Search strings were developed for each database, using Boolean operators to combine concepts. Because of the action focus, the search strategy was to include issues, interventions, and potential outcomes, which are represented in the three columns.

**Figure 1:**
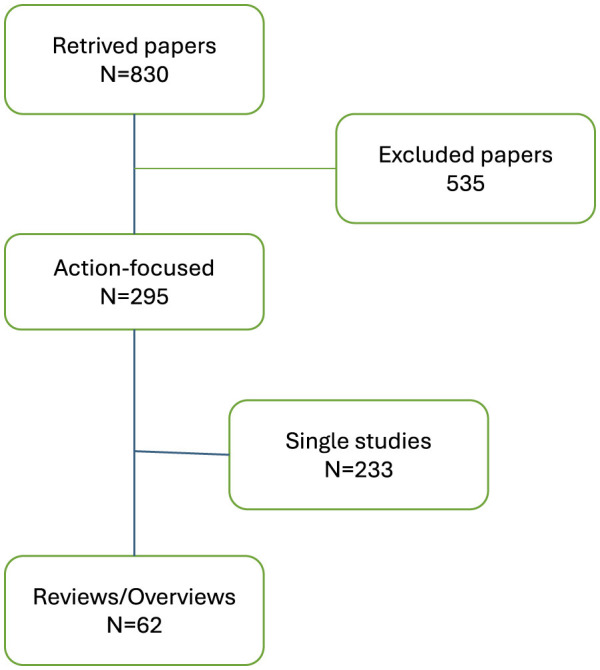
Flow chart of paper selection.

A total of 830 papers were identified, out of which 295 were retained owing to the focus on action (Figure 1). Based on abstracts, we excluded papers only describing health inequities, study protocols, research only papers, and articles without full online access. The first author selected the papers and validated them with at least one research team member. The 295 selected papers included 233 individual studies and 62 reviews. The present scoping review covers these review papers, whether standard reviews (systematic, narrative, integrative or scoping reviews) or overviews based on the literature. We defined “overviews” as broad descriptive summaries based on the literature but without a strict methodology, which is at variance with “standard” reviews.

The scoping review method followed the stages of the framework by Levac, Colquhoun and O'Brien: Identifying the research question; identifying relevant studies; study selection; charting the data; collating, summarizing and reporting the results; and consultation (15). The WHO building blocks for assessing health system performance (16) were adapted to classify the reviews into five key areas: Governance and policy; information and evidence data; technologies; human resources and capacity building; and health-related service delivery. The table used to extract the data from each selected review paper was developed by the research team (see Table 1). A narrative overview of the breadth of the initiatives reviewed under each key area is presented in the results, followed by a thematic discussion leading to recommendations to link one health and health equity actions.

**Table 1:** Summary of studies included in the review.

Authors and title	Objectives	Theme/focus	Methods	Findings	Recommendations by authors
Governance and policy					
Arcaya et al. (17) Neighborhoods and health: Interventions at the neighborhood level could help advance health equity	1) To critically review the relationships between neighborhoods and health; 2) to discuss policy responses	Social determinants of health (SDH): neighborhoods (USA)	Overview without a defined methodology	Neighborhood can contribute to health and wellbeing in different ways: 1) Institutions, e.g., number and quality of healthcare facilities 2) Physical characteristics, which affect safety, ability to engage in physical activity, access to healthy foods 3) Social conditions, e.g., violence, networks, segregation	Policy responses include better enforcement of fair housing laws, reforms to land use, and housing choice vouchers. Other measures to advance health equity include policy change to strengthen communities' social and physical infrastructure, and in particular, community-led initiatives to change material and social conditions, with explicit or non-explicit health promotion goals.
Jindal et al. (19) Policy solutions to eliminate racial and ethnic disparities in childhood in the USA	To critically review policies that reinforce and perpetuate health disparities in children, and key policy solutions	Social determinants of health: housing, economic opportunity and employment, health insurance, the criminal legal system, and immigration (USA)	Review without description of the search method. For each social sector, landmark articles are stated and examples of inequities are provided.	Housing quality, cost and segregation all have an impact on children's opportunities and health. Black and Asian children have less social mobility. Racial income gaps persist across generations unless targeted interventions are implemented. The US justice system is racially biased, including for youth. Immigrant-related policies which increase eligibility for employment, education and access to resources, termed inclusive policies, have been associated with better pediatric health outcomes.	There is a great need for political will to improve racial/ethnic child health equity. Investing in the SDHs could improve safety and reduce incarceration, which is racially biased. The funding of Immigrant-led and community organizations that support immigrant communities, providing language, food, legal, after-school, and health services, should be prioritized.
McMillan-Boyles et al. (22) Representations of clinical practice guidelines and health equity in healthcare literature: an integrative review	To explore how equity is discussed in the health literature in relation to clinical practice guidelines (CPG)	Clinical practice guidelines	Integrative review, with literature search in PubMed, CINAHL, Cochrane, EMBase, Medline, and Web of Science. Equity in CPG development, implementation and evaluation was documented.	139 papers published between 2010 and 2022 were screened and 19 were included in the review. CPGs can exacerbate health inequities if the resources and services outlined are not readily available and accessible. How health equity is integrated into CPG development is problematic, and the use of existing tools and checklists is challenging.	Equity should be clearly articulated into CPG at the outset of their development and throughout the different phases. Otherwise, the ability of health care professionals to implement the CPGs effectively and provide equitable health services may be hindered.
Shaver et al. (23) Health equity considerations in guideline development: a rapid scoping review	To synthesize current best practices for integrating health equity into guideline development, and the benefits or drawbacks of these practices	Guideline development	A “rapid” scoping review, with some components of the scoping review omitted or simplified. Scientific and gray literature papers were screened. The results were organized in four phases of guideline development: planning, evidence review, development and dissemination.	26 articles proposed best practices for incorporating health equity within guideline development. Equity guidance strategies were available for all four phases, and advantages and disadvantages were summarized. There are too many exemplary practices to summarize them here. Gaps were identified, for instance, no equity related guidance was captured to identify or report on conflicts of interest.	Guideline developers should consider the use of guideline checklists and tools to implement health equity promoting practices throughout guideline development. Any equity framework or plan should be developed in partnership with experts in the field of health equity, as well as health system stakeholders and community organizations.
Lee-Foon et al. (20) Saying and doing are different things: a scoping review on how health equity is conceptualized when considering healthcare system performance	To explore how healthcare systems around the world conceptualize equity when considering healthcare system performance.	Healthcare system performance	Scoping review using Levac approach was to identify relevant articles and develop a protocol. The peer-reviewed articles included were published between 2015 and 2020. Oral health and clinical training were excluded.	Of the 16 studies included, six were from North America, six from Europe and one each from Africa, Australia, China and India. Quantitative or qualitative studies centered on: indicators; equity policies; evaluating the equitability of healthcare systems; creating and/or testing equity tools; and using patients' sociodemographic characteristics to examine healthcare system performance. The definitions of equity varied widely, ranging from no definition to distributional fairness of healthcare services to populations with differing levels of disadvantage. All papers acknowledged that social determinants of health affected patients' health and their outcomes in various healthcare settings.	More research is needed to better understand the lack of consensus and how to ensure researchers are truly assessing the state of equity in their respective healthcare systems. More research should also be conducted to see if the impact of racism is being captured when assessing healthcare system performance. Additional resources are needed to address many of the social determinants of health that healthcare systems may have the capacity to address
Marcus et al. (25) Characterizing organizational health equity capacity assessments for public health organizations: a scoping review	To identify and characterize existing organizational health equity capacity assessments (OCAs).	Health equity performance assessment	Scoping review using the standard framework: identifying the research question and relevant studies; selecting studies; charting the data; collating and summarizing the results; and validating the findings with practitioners.	17 OCAs were included. Most aimed to provide considerations or strategies to increase organizational health equity capacity or readiness. These can be used repeatedly to monitor progress. Many OCAs lack specific definitions of organizational health equity capacity.	Future publications and case studies should include data related to OCA implementation, including capacity findings, implementation lessons learned, and resources required, where possible. Contextual information should be included in OCA publications, if possible.
Thornton et al. (21) Addressing population health inequities: investing in the social determinants of health for children and families to advance child health equity	To provide a critical assessment of recent pediatric population health research with a specific focus on child health equity, and to address the role of the healthcare sector in addressing fundamental social drivers of health.	Addressing the social determinants of health (SODH)	Overview without a defined methodology	The overall emphasis in the SDOH literature in pediatric populations focuses on the processes surrounding implementation: screening, uptake, response, and referral. What is less clear is whether screening accurately identifies social needs or improves overall health outcomes. Healthcare system efforts to identify and address individual needs are important but approaches centered on the individual level put the burden on patients and caregivers to disclose unmet needs in healthcare setting.	Screening and responding to urgent health related social needs and changing the social conditions that impact population health require a closer examination of systemic factors that produce certain patterns of disparities within the overall population. Social needs interventions need to pay attention to systems and structures and to explicitly recognize race and racism as key social determinants of health. Significant investments are needed in affordable housing, early childhood and universal pre-school programs, mental health support, and other upstream contributors to health outcomes. Researchers should partner with community organizations and policymakers to address root causes of health inequities. Value-based payment innovation to address SDOH has the potential to move investments upstream.
Reichman et al. (28) Using rising tides to lift all boats: equity-focused quality improvement as a tool to reduce neonatal health disparities	1) To provide a broad overview of neonatal health disparities scholarship; 2) To review the potential impact of Quality Improvement (QI) work on health disparities; and 3) To provide a framework for centering neonatal QI endeavors around equity.	Improvement of quality of care	Overview without a defined methodology	Infants of color experience significantly higher rates of low birthweight, preterm birth, and neonatal mortality compared to White infants. Geographic location also plays a role, with disparities in mortality and neonatal care quality observed between urban and rural areas, and even within different neighborhoods of the same city. The quality of care varies significantly between hospitals, contributing to disparities. Black and Hispanic infants are more likely to receive care in lower-quality hospitals, which exacerbates outcomes. There are also significant differences in neonatal mortality rates among different Asian subgroups and Hispanic subgroups, which are often overlooked. Other parental attributes may also contribute to disparities but are under-researched in neonatal care. QI methodologies can inadvertently exacerbate disparities if not explicitly designed to focus on equity.	Eight foundational concepts for designing and executing Equity Focused Quality Improvement (EF-QI) projects: 1. Foster a culture of equity 2. Identify the disparities 3. Incorporate equity in QI design 4. Involve families and community partners 5. Consider alternative comparator groups 6. Focus on root causes 7. Adapt data visualization tools 8. Disseminate data with equity in mind.
Plamondon et al. (18) Connecting knowledge with action for health equity: a critical interpretive synthesis of promising practices	To critically examine promising and empirically-derived strategies for advancing productive action on the root causes of health inequities.	Multi-disciplinary practices	Integrative review. A critical interpretive synthesis of empirical studies and literature reviews published whose authors framed health inequities as having known causes.	16 papers were included. Four groups of promising practices were identified: (re) structuring systems, working relationally, doing research, and carrying out knowledge translation. Restructuring systems involves the explicit analysis of power. Deploying social determinants of health nurses within the healthcare system was a key determinant of the efficacy and direction of the health equity work. Working relationally means fostering inclusion and connectedness, and mitigating power imbalances. In research, a promising practice was identified as embracing complexity in health equity work.	Cross-sectoral partnerships are needed since many structural determinants of health lie outside the health sector. Such partnerships also need to be studied: there is a gap in the literature. There is a need for capacity to recognize how societal structures, including dominant social values such as individualism and bio-behaviorism can promote actions that are directly in conflict with the evidence about root causes of health inequities.
				Regarding knowledge translation, blending numbers with human experience was deemed promising. However, there were few studies examining how to advance health equity at an institutional or societal level.	
Jensen et al. (24) Health equity and health system strengthening – time for a WHO re-think	1) To provide a critical reading of key policy documents and secondary literature to trace the conceptual and normative development of health equity as a guiding principle of WHO; 2) To highlight the limitations in the current conceptualization of equity in the framework of health system strengthening at the WHO.	The concept and practice of health equity	An overview without a defined methodology	The authors argue for the importance of re-considering what health equity implies in the context of health systems strengthening (HSS). Tracing the conception of equity at key periods in WHO's history, they cautioned against increasingly unidimensional conceptions of equity; as being a problem of either unequal access to specific healthcare services, or the differential health impacts of specific health interventions. They argue that a HSS agenda that focuses predominantly on improving health service delivery falls short of considering the structural political, social and economic dimensions that drive and sustain ill health and health inequities worldwide.	The authors point to potential avenues of interrogation: 1. The need to replace simplistic standardized frameworks to measure equity with broadened frameworks that identify intersecting forms of social disadvantage in particular contexts. 2. A first step in rethinking equity could involve addressing the equity questions that arise in relation to health workers in the context of HSS. 3. The need to re-focus attention onto the imbalances in resources and power and forms of oppression that undergird health inequities—and shape the global health field itself.
Sumah et al. (26) The impacts of decentralization on health-related equity: a systematic review of the evidence	To review the implications of decentralized governance of health care on equity in health, health care and health financing	Equity in health and healthcare	A systematic review that examined entire health systems and the relationship between implementing decentralized governance and health-related equity. The quality of reporting of the included studies was assessed.	Only nine papers (out of 808) met the inclusion criteria. The included studies were mostly explorative and used a range of quantitative techniques to analyze the relationship between variables of interest. The impact of decentralization on inequities in health and health care depends on pre-existing socio-economic disparities and financial barriers to access. While decentralization can lead to inequities in health financing between sub-national jurisdictions, this is minimized with substantial central government transfers and cross subsidization. The effect of decentralization on health-related equity can be best characterized as mixed.	The need for central coordination in decentralized health systems is paramount to define health system goals and outline the broad framework for their achievement when designing policy interventions. Equally important is the need for mechanisms to redistribute income to assuage disparity in financing health care between regions. Further research should look at comparative country study of decentralized and centralized national health systems.
Burström et al. (27) Equity aspects of the Primary Health Care Choice Reform in Sweden – a scoping review	1) To review the existing evidence of the impact of a recent Primary Health Care (PHC) Choice Reform on health equity; 2) To Identify the gaps in the current literature.	PHC policy reform	A scoping review	The studies to date indicate that the PHC Choice Reform, as implemented, increased access to PHC and increased the average number of visits to PHC, but seems to have particularly benefited those in more affluent groups and with lower health care needs. The PHC Choice Reform has made integrated care for those with complex needs more difficult.	The PHC Choice Reform may have damaged equity of primary health care provision, contrary to the tenets of the Swedish Health and Medical Service Act. This situation needs to be carefully monitored. Further studies are needed to follow up on the long-term impacts of the reform on the structure, process, and outcomes of PHC in Sweden and how different types of reimbursement systems may modulate these impacts.
Spitzer-Shohat and Chin (29) The “Waze” of inequity reduction frameworks for organizations: a scoping review	1) To identify existing frameworks focused on reducing inequities in patient care and outcomes; 2) To assess to what extent the frameworks address key organizational change elements.	Tools for organizations to become more equitable	A scoping review. The analyses were conducted on context, processes, outcomes and time, that is, the four constructs of organizational change.	14 frameworks and models were analyzed, all of them from rich countries. They were developed by governments, healthcare associations, not-for-profit associations, and academia. Most frameworks did not guide the translation of equity across multiple organizational departments and levels. Existing equity intervention frameworks often lack specific guidance for implementing organizational change. Most frameworks primarily focused on the organization's outer context through the analysis of data on race and ethnicity. Two frameworks recognize the importance of existing culture: the CLAS (Culturally and Linguistically Appropriate Services Standards), and the Disparity Leadership Program. Most frameworks address the implementation process at the macro level, but the Roadmap to Reduce Disparities model offers change directives not only on the macro level, but also the meso and micro levels.	Providing organizations clear, effective, and concrete guidance has great potential from improving health equity. Frameworks should include guidelines on assessment of inner organization context parameters such as readiness for change. Organizations also require specific guidance on how to implement equity within and across all organizational levels. Guidance for institutionalization and sustainability are crucial. Future frameworks should assess the inner organizational context to guide the translation of programs across different organizational departments and levels and provide specific guidelines on institutionalization and sustainability of interventions.
				Regarding outcomes, the Achieving Health Equity framework suggests measuring performance for individual socio demographic attributes. The measurements are combined into a summary index, which is then compared with the best health level among all groups as reference.	
2 Information and evidence data					
Hirsch et al. (30) Evidence clearinghouses as tools to advance health equity: what we know from a systematic scan	To explore how clearinghouses communicated an intervention's health equity impact and to review their health equity definition and underlying methods.	Informing on health equity impact	Scoping review: a systematic scan, a comprehensive directory of clearinghouses and a comparative analysis of clearinghouses with publicly available health equity impact reviews on their websites.	The authors identified 18 clearinghouses that were USA-focused, web-based registries of interventions that assigned an effectiveness rating for improving community health and the social determinants of health. Only seven clearinghouses summarized an intervention's potential impact on health equity. However, they defined and operationalized equity differently, and most lacked transparency in their review methods. One or more approaches were used to communicate the findings: summarize study findings on differential impact for subpopulations, curate interventions that reduce health disparities, and/or assign a disparity/equity rating to each intervention.	Advancing equity through an evidence- informed approach will require researchers to conduct more equity-focused research and clearinghouses to evolve as practice-oriented tools with health equity impact reviews based on clear and transparent underlying definitions, values and methods
Hollands et al. (31) Methods used to conceptualize dimensions of health equity impacts of public health interventions in systematic reviews	1)To identify and summarize methods, frameworks, and tools used as a conceptual basis for investigating dimensions of equity impact of public health interventions;	Assessment of health equity impacts	An umbrella review of systematic reviews with a focus on the equity impacts of public health interventions.	The majority of reviews originated from European countries, especially the UK. 37.5% used PROGRESS-Plus. Some reviews adapted PROGRESS-Plus with additional dimensions linked to equity.	Primary studies need to collect and report equity-related data consistently. Support from research funders, regulators, and scientific journals is necessary. Standardized guidelines
	2) To document challenges and opportunities encountered in the application of such methods, as reported in the systematic reviews.		Electronic searches of the Cochrane Database of Systematic Reviews, the Database of Promoting Health Effectiveness Reviews (DoPHER), the Finding Accessible Inequalities Research in Public Health Database, and the automated searches of the Open Alex dataset.	Planned methods for conceptualizing equity impacts were fully applied in less than half of the reviews. The primary reasons for the incomplete application were the lack of necessary information in primary studies, a lack of included studies, inadequate study quality, and low heterogeneity by key dimensions. Measurement issues related to dimensions of equity impact were a notable problem, the primary concern being the difficulty of investigating constructs that lack standardized definitions, operationalization, and ultimately measurement, particularly for socioeconomic status and closely related concepts.	and practical guidance are needed to operationalize and analyze equity dimensions consistently. Broader conceptual frameworks, such as socioecological models, may better capture complex and intersecting pathways of inequities. More explicitly rationalized and considered approaches to the design, conduct, and reporting of primary research and systematic reviews are necessary to address these challenges.
Hosseinpoor et al. (32) Strengthening and expanding health inequality monitoring for the advancement of health equity: a review of WHO resources and contributions	1) To review WHO's work on health inequality monitoring; 2) To demonstrate how this multi-faceted strategy and associated resources can accelerate health inequality monitoring practices among Member States and raise the profile of global evidence on health inequalities.	Health inequality monitoring	A synthesis review of various strategies, resources, and tools developed by WHO including the overview of the 2022–2027 Inequality Monitoring and Analysis Strategy, specific goals, activities, and resources: manuals, workbooks, eLearning courses, workshops, and software applications like the Health Equity Assessment Toolkit (HEAT).	The Inequality Monitoring and Analysis Strategy has three goals: strengthening the capacity for health inequality monitoring; generating and disseminating high-quality evidence on health inequality; and developing and refining health inequality monitoring methods, tools, resources, and best practices Guiding material for health inequality monitoring includes manuals and accompanying workbooks, the Health Inequality Monitoring eLearning channel, and capacity building workshops. HEAT and HEAT Plus is a free and open-source software application that facilitates the assessment of within-country health Inequalities using disaggregated data. The Health Inequality Data Repository is the largest collection of publicly available disaggregated data about health and its determinants.	To use the evidence generated from health inequality monitoring to inform and guide policy changes and program improvements. To utilize WHO resources and tools to improve data collection, analysis, and reporting. To use WHO's eLearning courses and workshops for continuous learning and capacity building. To encourage the integration of health inequality monitoring into routine health information systems and research initiatives.
Pearson et al. (37) Climate change and health equity: a research agenda for psychological science	1) To examine the role of climate change as a unique source and magnifier of health inequities; 2) To consider mediating psychological processes that may fuel and magnify health inequities; and 3) To consider the infrastructure needed to speed the development and adoption of science and community-informed solutions, including perspectives of communities.	Climate change and health inequities	Overview without a defined methodology. The authors expand on two existing frameworks (social vulnerability, and direct and indirect effects of climate change on health and wellbeing).	Globally, small island nations and indigenous communities are among the most affected by climate change. At the same time, these communities play a central role in managing earth's ecosystem. Inequities can stem from both adaptation and are designed without considering or including vulnerable groups. Three systemic factors shape climate health equity: structural racism, segregation and displacement. Misperception of climate health risk and social vulnerability is an additional factor.	The intersection of climate change and health inequities presents new challenges and opportunities for health intervention that will require new research infrastructure, collaborations, and training initiatives. Climate interventions that address existing inequities may be more effective in mitigating climate change than those that fail to take health inequities into account. Highlighting cobenefits of climate measures that improve health outcomes and reduce inequities can help secure public support for climate action. A “whole-of-science” approach is needed to address climate-related health inequities.
Cené et al. (34) Racial health equity and social needs interventions a review of a scoping review	To understand how studies of interventions addressing social needs among multiracial or multiethnic populations conceptualize and analyze differential intervention outcomes by race or ethnicity	Social needs interventions	A rapid scoping review, with specific methodological adjustments: reliance on existing searches for the evidence map; no second review of the risk of bias; single reviewer recheck of data for subgroup or effect modification analyses; focused data extraction outcomes; no strength of evidence grading; and a primarily narrative or qualitative synthesis. The focus was on studies in multiracial or multiethnic populations to examine differential intervention outcomes by race or ethnicity.	The review used a simple framework of conceptual thoughtfulness and analytical informativeness to understand how social needs interventions may advance racial health equity. Among 152 studies, 44 (28%) included race or ethnicity in their analyses. Only 9% of the studies were considered conceptually thoughtful, explaining race as a proxy for exposure to racism. Few studies (21 [14%]) conducted race or ethnicity–stratified analyses that were considered analytically informative for advancing health equity research, with 14 reporting no differences. Of the 7 that did report differences, 3 had mixed outcomes and 4 found interventions benefited minoritized populations more and provided conceptually thoughtful explanations for race as a proxy for root causes of racial health inequities. Nearly 9	Continued education on the need to provide theory-driven conceptualizations of race and ethnicity, the risks of not doing so, and standard guidance on where such descriptions should be provided. The proposed innovative two-concept framework for assessing a study's contribution to racial health equity (conceptually thoughtful, analytically informative) should be Incorporated into standards for systematic reviews on health equity. Journals should revise instructions to emphasize handling race and racism from conceptualization through data analyses and interpretation.
				in 10 (86%) of the 152 studies in multiracial or multiethnic populations did not examine whether intervention effects differed by race or ethnicity.	
Garrett et al. (35) Antibias efforts in United States maternity care: a scoping review of the publicly-funded health equity intervention pipeline	1) To investigate whether recent national public funding reflects the heightened priority of the increase in public and governmental support for antibias and antiracism interventions; 2) To identify and characterize publicly funded interventions designed to reduce bias, racism, and discrimination among maternal healthcare providers in the United States.	Reducing bias and racism in maternal healthcare	A rapid scoping review. The search for publicly funded grants was conducted in the Dimensions database, a comprehensive registry of federal, public/private, and large philanthropic grantees.	Only four of 508 projects met the search criteria, featuring an intervention to reduce bias or racism in maternal healthcare providers. One of the projects proposes a “racial equity training” for perinatal care clinicians to benefit Black women receiving prenatal care. A second project proposes to deliver antiracism training to medical providers to reduce Black and African American patients' experiences of racism or mistreatment and promote respectful maternity care. A third project proposes a five-year, multilevel intervention co-developed with community partners to reduce the rate of maternal morbidity and mortality among Medicaid-insured African American women by intervening at various levels, including antibias training at the provider/practice level for physicians, midwives, hospital administrators, and front desk staff. The fourth project implements an “interactive racial equity training” designed to help prenatal clinic staff to recognize their implicit biases and understand how racism affects pregnancy care for patients of color.	The reviewed projects employ promising and innovative components such as community-based participatory research, but there is little material in support of intervening on racial bias. Several gaps were identified, e.g., how best to develop and implement bias training, and what is their impact on patient outcomes. Philanthropically funded and community-grounded work will be important to help bridge this knowledge gap. Large funders should support iterative national reviews of emergent research and convene multiple sectors—including policymakers, payers, providers, community members, and patients—to align interventions and policies with new evidence while centering on the needs of Black women, birthing people, and others harmed by bias and racism in the healthcare system.
Ramanadhan et al. (33) Using participatory implementation science to advance health equity	1) To provide guidance on the principles and practice of participatory implementation science (IS); 2) To introduce readers to the value of participatory approaches for strengthening sustainable implementation of health-related evidence-based interventions; 3) To provide a framework for applying the principles, practices, and lessons from participatory research to IS; and 4) To outline challenges and considerations for optimizing the potential of participatory IS.	Participatory approach to implementation science	Overview without a defined methodology	Traditional IS often lacks a focus on health inequities and typically uses a top-down approach, whereas participatory IS emphasizes iterative co-creation of knowledge and action, integrating diverse perspectives, including those from lived experiences. The participatory approach enables researchers, community members, and other relevant actors to work together, generate knowledge and drive change. It also decentralizes dominant perspectives to address health inequities and explicitly engages with issues of power and representation to facilitate the meaningful participation of marginalized groups in creating transformational knowledge and change. It focuses on relevant evidence, deeper understanding of local contexts, and building capacity and solutions for health issues. Additionally, it values the research process and aims to advance justice, inclusion, and equity. Participatory IS moves beyond making EBIs work, deploying implementation efforts to reshape systems and intervention contexts in ways that center equity. However, participatory IS requires time and resources, and engagement.	To optimize participatory Implementation Science (IS): Assessing team readiness and engaging in critical reflexivity considering how team members' roles and social positions impact research—is crucial; Navigating tensions between world views within teams to prevent frictions and misunderstandings; Aligning projects with broader perspectives; And developing measures to advance the evidence base for participatory IS.
3 Technologies and tools					
Rojas-Rueda et al. (45) Addressing health equity in the context of carbon capture, utilization, and sequestration technologies	1. To identify recent findings related to the implementation of carbon capture, utilization and sequestration technologies (CCUS) and their impacts on social determinants of health; 2) To discuss the challenges and opportunities related to health equity.	Climate change technologies	Overview without defined methodology.	CCUS technologies have the potential to both improve and worsen health equity. They could help reduce greenhouse gas emissions, a major contributor to climate change, but they could also have negative health impacts like air and noise pollution.	Efforts to reduce carbon emissions should prioritize the needs and perspectives of the most vulnerable populations and ensure that the benefits and burdens of carbon reduction policies not only are distributed equitably but also contribute to restoring relationships with and opportunities for historically marginalized groups. The effective deployment of CCUS technologies requires a critical assessment of their potential impacts on public health and environmental equity. Decision-makers must confirm aggressive climate mitigation policies are already in place before considering CCUS as part of a comprehensive emission reduction strategy.
Rabet et al. (38) Barriers and facilitators to digital primary health care access in immigrant and refugee populations: a scoping review	To identify: 1) The barriers and facilitators for access to digital primary health care among immigrants and refugees; 2) The primary health care needs addressed through digital modalities	Primary health care access through digital health technology	Scoping review of qualitative studies in high and in low or middle-income countries. Levesque's model (70) was used to examine approachability, acceptability, availability/accommodation, affordability and appropriateness.	25 papers were included. The flexibility of digital modalities was a facilitator, but older age or limited digital literacy skills were barriers. Social networks (family, community) were important to support this access. Immigration systems play a role by affecting living conditions and financial means of these groups. Privacy and data security are major concerns and can be important barriers for these groups	Social assistance programs and affordable housing schemes can provide refugees and immigrants with safe living arrangements, income support and digital technologies. Providing digital literacy programs and use of cheaper and accessible forms of technology such as text messaging and audio-calling are also recommended. Research should explore how the personal data of those with precarious status are managed and develop guiding principles for digital health applications among these groups.
Cary et al. (46) Mitigating racial and ethnic bias and advancing health equity in clinical algorithms: a scoping review	To review health care applications, frameworks, reviews and perspectives, and assessment tools that identify and mitigate bias in clinical algorithms, with a specific focus on racial and ethnic bias.	Discrimination and bias	Scoping review of tools, frameworks, reviews and perspectives on bias mitigating strategies	109 studies were included; reviews and perspectives were the most frequent type. Several mitigation strategies were identified. The technical strategies pertained to various stages of the algorithm development process. The operational strategies included governance, design principles and the engagement of multidisciplinary teams. System-wide strategies included training and education on risk of bias, collaborative platforms, and development of standards. No consensus on a single best practice was found.	To ensure professional diversity; To require auditable clinical algorithms; To foster transparent organizational culture; To implement health equity by design; To accelerate research; To establish governance structures; and to amplify patients' voices. Future research should identify optimal bias mitigation methods for various scenarios, depending on factors such as patient population, clinical setting, algorithm design, and types of bias to be addressed.
Fisher et al. (48) Occupational safety and health equity impacts of artificial intelligence: a scoping review	To summarize the recent literature on the way in which Artificial Intelligence (AI) can reduce or exacerbate inequities in occupational safety and health (OSH).	Artificial intelligence	Scoping review adapting PRISMA and designed around three concepts: artificial intelligence, occupational safety and health (OSH), and health equity. Research questions were: how can AI be used to promote OSH equity? How does Ai present barriers and challenges to OSH equity? What are best practices to address emerging OSH equity challenges related to AI?	112 papers were included. Certain communities take on a higher burden of dangerous work and traumatic injuries (in construction, transportation, mining). By reducing exposure to hazardous conditions in these industries, AI has the potential to reduce occupational health inequities for workers from these communities. Algorithmic integrity in the form of proper systems to curb the mishandling and misuse of received data is necessary in order to reduce bias. The digital divide mainly affects individuals from low-resourced communities. Increases in depression, suicide, and alcohol and drug abuse, including opioid-related death, may occur, exacerbating health inequities.	An ethical code or framework for justice in AI development and implementation was frequently recommended in the literature, and would facilitate OSH equity. AI's role in OSH equity is vastly understudied. An urgent need exists for multidisciplinary research that addresses where and how AI is being adopted and evaluated and how its use is affecting OSH across industries, wage categories, and sociodemographic groups. OSH professionals can play a significant role in identifying strategies that ensure the benefits of AI in promoting workforce health and wellbeing are equitably distributed.
Petretto et al. (39) Telemedicine, e-health, and digital health equity: a scoping review	The research questions were: How did previous papers: 1) Define and describe digital health equity (DHE) in telemedicine and e-health; 2) Describe barriers and risk factors in the promotion of DHE in those e-environments; 3) Describe the advantages of the use of telemedicine and e-health for the promotion of DHE; and 4) Describe ways to improve equity in e-health and telemedicine	Digital health	A scoping review using the PRISMA-ScR guidelines	31 papers were included: editorials, commentaries, viewpoints and only a few research papers. An interesting one is the distinction of 3 levels of digital divide: lack of access, lack of skills, and lack of possibility to use the tools for one's health. The digital divide or disadvantage is the of the interaction between any person and an environment that is “not sufficiently equipped to promote promote health equity”. The role of telemedicine and e-health in reducing the gap in access to services is widely recognized. The digital and the social determinants of health interact to increase or reduce digital health equity.	It is useful to list the reasons/variables that can facilitate the occurrence of the condition of disadvantage. There is a need to have an overall and integrated picture of all these variables, a multilevel complex model of “telemedicine and e-health ecosystem.” Government, scientific societies, stakeholders, and health policymakers may have a central role in planning and implementing specific interventions to promote digital health equity, providing system-level changes according to the chosen multilevel complex model.
Sharrief et al. (40) Telehealth trials to address health equity in stroke survivors	1) To review the telehealth advantages and barriers for the chronic care of stroke survivors; 2) To discuss strategies to address barriers to telehealth use in stroke patients with adverse social determinants of health	Telehealth (or tele-medicine) for stroke care	Review without description of the method	Telestroke has increased access to acute stroke care for populations at risk for poor outcomes. However, the use of telehealth applications for expanding access to other aspects of stroke patient care and for reducing disparities in stroke outcomes has been under-studied. Telehealth for the ambulatory care of various chronic diseases (heart failure, diabetes, Parkinson's disease, neurological diseases) was usually found to be as effective as in-person care. However, the results of telehealth care on health inequities among groups at higher outcome risk has not been studied.	Telehealth for transitional and chronic care of stroke survivors with a higher proportion of adverse social determinants of health (economic instability, low educational attainment, low health literacy, and low levels of social support) may help to address access- related issues and therefore holds promise for addressing disparities in stroke outcomes. However, the need for a patient to access care via telehealth requires digital literacy, consistent telephone and internet access, and increasingly, the ability and willingness to engage with the electronic medical record through patient portals. There are also health system barriers, such as billing restrictions. it is essential that systems be built with health equity in mind.
Vakkalenka et al. (42) Telehealth use and health equity for mental health and substance use disorder during the COVID-19 pandemic: a systematic review	1) To evaluate health equality in clinical effectiveness and utilization measures associated with telehealth for clinical management of mental health disorders and substance use disorders; 2) To identify under-represented groups.	Digital health for mental disease and drug abuse	Systematic review using relevant elements of PRISMA guidelines. Studies on tobacco cessation only or neurocognitive conditions (e.g., dementia, Alzheimer's disease, autism) were excluded. Overall, the most common dimensions captured included race/ethnicity or gender. Risk of bias was also assessed.	25 studies were included, 20 of which on mental health. All 25 evaluated synchronous, direct-to-consumer video telehealth. These conditions in themselves reflect underserved and marginalized populations. Most studies identified that telehealth implementation suffered from significant and widening disparities for disadvantaged populations, including rural populations, older patients, and racial/ethnic minorities.	If the technological vehicle used to address inequities further propagates a digital divide, policymakers should examine individual-, innovation-, and system-level implementation processes and policies that promote or hinder equity in adoption, utilization, and clinical effectiveness. Future efforts should focus on measuring the contribution of utilization disparities on outcomes and strategies to mitigate disparities in implementation.
Campanozzi et al. (44) The role of digital literacy in achieving health equity in the third millennium society: a literature review	To assess the extent of the impact of digital literacy on access to telemedicine services	Access to digital health services	Review of papers 2011–2022. Databases for the gray literature were omitted.	37 articles were included. The importance of digital literacy for the equitable distribution of health services in the third millennium is recognized. Ensuring equity of access to digital health must be a priority felt by the various stakeholders. It is essential to develop screening tools that can accurately identify the population groups in need of digital literacy interventions.	It is not only important to implement digital education programs that can bridge as much of the “digital divide” as possible, but it is equally important to plan for evaluation studies of the effectiveness of such programs in the immediate future.
Hynie et al. (43) Access to virtual mental healthcare and support for refugee and immigrant groups: a scoping review	To explore the potential of increased access through virtual mental healthcare services (VMHS) for these populations using the patient-centered model of Levesque (74)	Access to virtual mental health care	A scoping review from November 2020 through October 2021.	2,561 abstracts were reviewed, 40 unique interventions identified. Studies include cultural adaptations, feasibility/pilot studies, usability studies, and formative evaluations.	The importance of more implementation research was highlighted. Unique barriers determined by systemic, contextual, clinical and personal characteristics for immigrant and refugee populations were identified. Such
			The authors investigated the accessibility (affordability, availability/accommodation, appropriateness, and acceptability) of virtual mental health services for immigrants, refugees, and asylum seekers.	Nature of Interventions: primarily mental health interventions, diagnostic assessment studies, screening tools, and user-testing of interventions. Delivery modalities were web/mobile apps primarily, video calls, phone interventions, tablet-based, and text-based. Accessibility depended on individual (e.g., literacy), program (e.g., computer required) and contextual/social factors (e.g., housing characteristics, internet bandwidth). Participation often required financial and technical support, raising important questions about the generalizability and sustainability of VMHS' accessibility for immigrant and refugee populations.	obstacles warrant further attention. It is proposed that working with the intended user population on the planning and delivery of virtual mental health services will help increase accessibility for these populations, both now and in the future.
Bakken et al. (41) Behavioral interventions using consumer information technology as tools to advance health equity	To demonstrate the use of mHealth, telehealth, and social media as behavioral intervention platforms in health disparity populations, to identify challenges to achieving their use, to describe strategies for overcoming the challenges, and to recommend future directions.	Consumer information technology (CIT) for behavior change	Umbrella review. Literature and case examples are summarized. A substantial number of systematic reviews and meta-analysis assessed the quality of intervention studies and the evidence across studies was synthesized, particularly randomized controlled trials, to advance CIT-enabled interventions.	The examples presented suggest that mHealth, telehealth, and social-media-enabled behavioral interventions, particularly the multicomponent interventions, show promise and in some instances influence health outcomes of interest in health disparity populations. The challenges in the design, implementation and evaluation of CIT-enabled behavioral interventions with health disparity populations are described.	Future directions include improved design methods, enhanced research reporting. advancement of multilevel interventions, rigorous evaluation, efforts to address privacy concerns, and inclusive design and implementation decisions, and to advance multilevel interventions by linking mHealth and social media-enabled interventions with healthcare delivery system. Also, to evaluate mHealth, telehealth, and social media-based interventions throughout the stages of developing and implementing the CIT-enabled intervention. Furthermore, to make design and implementation decisions that foster the inclusion and sustained engagement of health disparity populations in CIT-enabled intervention studies, and to address user privacy concerns.
Siddique et al. (47) The Impact of health care algorithms on racial and ethnic disparities: a systematic review	To examine: 1) The evidence on whether and how healthcare algorithms (aggravate, perpetuate, or reduce racial and ethnic disparities in access to healthcare, quality of care, and health outcomes; and 2) Strategies that mitigate racial and ethnic bias in the development and use of algorithms	Rrcial and ethnic disparities in health	Systematic review using predefined criteria to assess one or both key questions: the effect of algorithms on racial and ethnic disparities in healthcare and outcomes, and the effect of strategies or approaches to mitigate racial and ethnic bias in the development validation, dissemination, and implementation of algorithms.	The review includes 63 studies in the USA, out of which 49 pertain to mitigating strategies. The most common algorithms evaluated kidney function and cardiovascular risk. Strategies involved removing, adding, adding, or changing variables. The algorithms can affect racial and ethnic disparities in health care (and outcomes) even if race and ethnicity are explicit inputs. Evidence suggesting that algorithms may disparities, perpetuate or exacerbate them, or not affect them was found. Most studies reported that mitigation strategies reduced racial and ethnic disparities in care. However, there were wide variations in the populations and diseases considered.	Investing in further research to assess the real-world effect of algorithms on racial and ethnic disparities before widespread implementation is recommended.
4 Human resources					
Adams et al. (49) Integrating nurse practitioners into primary healthcare to advance health equity through a social justice lens: an integrative review	1) To develop a framework to guide the successful integration of nurse practitioners (NPs) into practice settings; 2) Working from a social justice lens, to deliver comprehensive primary healthcare which advances health equity	Workforce issues	An integrative review. PRISMA guidelines were followed. Data were extracted and thematically analyzed using NVivo.	28 articles were included. Six themes were identified at the individual (micro), local health provider (meso), and national systems and structures (macro) levels of the health sector: (1) autonomy and agency; (2) awareness and visibility; (3) shared vision; (4) leadership; (5) funding and infrastructure; and (6) intentional support and self-care. Based on this the authors developed a framework to guide the integration of NPs into PHC.	The proposed framework is to support the integration of NPs into PHC settings where they can optimize their scope of practice and deliver healthcare services that improve healthcare access and health outcomes to achieve equity. The framework should be tested in practice in a range of settings and adapted to meet the meet the local context, community needs and the NP workforce capabilities. Working with communities co-designing health service delivery with other health and social agencies is critical if local community health needs are to be met and disparities eliminated.
Graefe et al. (50) Advancing health equity in prelicensure nursing curricula: findings from a critical review	To determine the extent to which health equity concepts are explicitly present in prelicensure undergraduate nursing curricula globally.	Workforce issues	Critical review. Health equity content was categorized based on the Commission on Social Determinants of Health (CSDH) framework categories	20 unique studies were reviewed. Frequency and quantity of health equity content, concepts topics, teaching strategies, evaluation strategies, and the overall extent of integration varied widely. Only two papers described overall well-integrated explicit health equity content, and there was little attention to whether students transferred this learning into practice. A focus on individualism rather than population and community was noted.	The findings suggest there is a gap (and need of) health equity content throughout the nursing curriculum. The authors argue that such an intervention could help nursing students understand the root causes of health outcomes beyond the individual and intermediary levels and prepare nurses to enter practice with a critical awareness of social and systemic health barriers (e.g., whiteness) and facilitators (e.g., community strength and healing) that influence health and illness. Additional content related to governance and policy, history and historic context, and cultural and social values is needed.
Chandler et al. (52) Training public health students in racial justice and health equity: a systematic review	To identify approaches, programs, pedagogical methods, and curricula that exist to support the training of US public health students in understanding racism as structural determinant of health.	Racism as a determinant of health	A systematic review of peer-reviewed literature. The Systematic Reviews and Meta-Analyses (PRISMA) guidelines were followed.	Only 11 examples of peer-reviewed articles were found on curricula, lessons and competencies developed to better understand racism as a structural determinant of health. Programs included workshop or seminars, and went from 90-min workshops to semester-long courses. Materials and resources included in-person presentations, music, artistic material, YouTube videos, a local museum, documentaries, television shows, and toolkits. Six out of the 11 programs included some form of evaluation. However, existing peer-reviewed literature provides little pedagogical guidance to inform schools on how to teach about racism and health equity.	There is little consensus on how best to teach about racism. More research on public health pedagogy pedagogy on structural racism is needed. Schools and programs of public health must explain the political, and economic determinants of health and how they contribute to population health and health health inequities. More systematic and rigorous approaches to public health pedagogy, including development of competency-based models and learning communities on evidence-based education, are recommended. More research is needed to document how to educate public health students on the health issues such as racial disparities they will address in their practice.
Ahmed et al. (51) Community health workers and health equity in low- and middle-income countries: systematic review and recommendations for policy and practice	1) To synthesize data on the effectiveness of community health workers' (CHWs) interventions at reaching more disadvantaged groups in low- and middle-income countries (LMICs); 2) To summarize the evidence on whether and how these programs reduce health inequities	Workforce issues	A review without description of the method. The “equity stratifiers” of the PROGRESS framework, such as race, gender, religion, social capital, etc., are used to assess the impact of CHWs' interventions on inequities.	167 studies were included, carried out in 33 LMICs; 72 were qualitative. Only eight studies were high-quality randomized trials. The results suggest that CHW programmes achieved greater equitability in service delivery than outcomes. Regarding service delivery, pro-equity findings outnumbered anti-equity findings across several stratifiers, but some marginalized groups are still being excluded. Pro-equity outcomes outnumbered the anti-equity ones only for gender and occupation; equitable service delivery did not delivery did not always translate into improved outcomes. CHW programmes may also influence health advocacy, investment of their personal resources, or hiring of CHWs in disadvantaged groups. However, they often have poor working conditions	To reduce inequities of access to health services, several recommendations are made according to major equity stratifiers, such as: -Place of residence: transportation for CHWs and patients; -Socio-economic status: food parcels as part of CHWs services and financial incentives for those most in need; -Gender: division of tasks between male and female CHW; -Education: the use of illustrated informative material to serve low-education people; -Minority race or ethnic groups: recruiting CHWs within the minority groups; -Social capital: CHWs accompanying the patient to the health facility or writing a referral slip; -Occupation: adjusting CHW schedule to fit with those of working patients. It is important to also consider intersectionality, for instance, gender intersecting with poverty and rural remoteness,
Curtis et al. (85) Why cultural safety rather than cultural competency is required to achieve health equity: a literature review and recommended definition	To redefine cultural safety to achieve health equity	Social determinants of health and health systems	A review of international articles on the definition of cultural competency and cultural safety published between 1989 and 2018. This review and analysis were conducted from an Indigenous research position that draws from Kaupapa Māori theoretical and research approaches	69 papers were included. Equitable care is further compromised by the paradox of well-intentioned physicians providing inequitable care: poor communication, a lack of partnership via participatory or shared decision-making, a lack of respect, familiarity or affiliation, and an overall lack of trust. Cultural safety requires health providers to question their own biases, attitudes, assumptions, stereotypes and prejudices that may be contributing to a lower quality of healthcare for some patients.	Health practitioners, healthcare organizations and health systems need to be engaged in working toward cultural safety as defined by patients and communities, and critical consciousness rather than narrow cultural competency. The objective of cultural safety activities also needs to be clearly linked to achieving health equity. Cultural safety activities should extend beyond formal training curricula or acquiring knowledge about other cultures. The framing of cultural safety requires a focus on power relationships and inequities within healthcare interactions.
5 Service delivery					
Lopez et al. (53) Achieving health equity in the care of patients with heart failure	1) To discuss the prevailing racial and ethnic disparities in heart failure (HF) care by identifying barriers to equitable care; 2) To propose solutions for achieving equitable outcomes	Service delivery, heart failure	A review without description of the method. From prevention to advanced interventions, current efforts are described and recommendations made for improvement.	Racial and ethnic disparities prevail throughout the entire spectrum of HF care, from prevention to implementation of guideline-directed medical therapy and advanced interventions. Factors such as differential distribution of risk factors, poor access to care, inadequate representation in clinical trials, and discrimination by healthcare clinicians, among others, contribute to these disparities.	This review emphasizes the importance of a multifaceted approach involving policy changes, quality improvement strategies, targeted interventions, and intentional community engagement. The authors proposed a framework integrating equity into routine quality improvement efforts, tailoring interventions to specific populations, and advocating for policy transformation.
Lopez et al. (54) Health equity and policy considerations for pediatric and adult congenital heart disease care among minoritized populations in the United States	1) To review the existing disparities among marginalized or racially minoritized populations with congenital heart disease in the USA and to propose solutions; 2) To critically examine multilevel factors and health policies that continue to drive health inequities, including varying social determinants of health (SDOH), systemic inequities, and structural racism.	Service delivery, heart failure	A review without description of the method. After documenting the causes of disparities throughout the lifespan of minoritized populations with congenital heart disease in the USA, potential solutions for the various minoritized populations taken separately are exposed. The review addresses system-level health policies that impact on reimbursement and research funding, as well as institutional policies that impact leadership diversity and representation in the workforce	This review describes the challenges facing various population groups with congenital heart disease: disease: Native, Black, Latino, LGBTQ and persons with disabilities. Disparities begin as early as care, with lower prenatal screening rates and poorer health outcomes among minoritized groups. Insurance status and maternal education play a significant role in these disparities. Disinvestment in marginalized communities leads to poorer education, income, and healthcare access, contributing to higher mortality rates and persistent health disparities in CHD populations. Conditions of reimbursement, including lower reimbursement rates for pediatric care, exacerbate disparities. Minoritized groups are underrepresented in medicine, particularly in pediatric cardiology, impacting patient care and health outcomes.	A wealth of solutions is proposed at the system and institutional level. Only those at system level are mentioned here. Solutions for American Indian/Alaska native (AI/AN) populations include: a policy to encourage Indigenous sovereignty, preserving culture, language, and community, and supporting the funding and structure of programs, including home visitation programs. Potential solutions for non-Hispanic Black populations include: improved neighborhood conditions, food assistance programs, and improved health literacy. Solutions for Hispanic/Latino populations are to expand Medicaid for children and provide translation services. For other groups: to prohibit discrimination in health insurance and integrate LGBTQ health content into medical curricula and ensure healthcare accessibility and support insurance coverage for persons with disabilities.
McNeill et al. (56) Uses of social determinants of health data to address cardiovascular disease and health equity: a scoping review	1) To explore what and how social determinants of health data are being used to address cardiovascular disease and improve health equity; 2) To identify gaps in evidence by focusing on the ways in which SDOH data have been applied to improve CVD outcomes, largely in the United States but also in other high-income countries.	Social determinants of health	A scoping review including studies published between 2014 and 2022, involving adult populations, and containing data related to SDOH and outcomes related to CVD.	The review included 50 articles and examined three broad domains of data, social determinants of health, and CVD. Practicing clinicians have called for the use of big data on SDOH to address CVD and health equity. The most common SDOH domain among the studies were healthcare access and quality, followed by the neighborhood and built environment. Few studies focused on economic stability, social and community context, or education access and quality. SDOH data have been used to understand the relationship between the built environment and CVD outcomes in 27 studies. The data were used to describe the prevalence or incidence of CVD risk factors and outcomes, and to create climate vulnerability maps. Other uses of the data were to evaluate social risk scores, and to develop interventions, including digital health applications for patient self-management and health literacy.	Healthcare providers, policymakers, and researchers should consider integrating multiple SDOH domains to develop interventions and improve CVD outcomes, including economic stability and social and community context, as well as the neighborhood and built environment, and education access and quality. More research is needed to measure and examine the role racism plays as a driver of cardiovascular health inequities. Combining a wide array of data sources, including non-health sector data, could provide a more comprehensive understanding of SDOH and CVD outcomes and help limit bias.
Lopez-Suarez et al. (62) A toolkit of health equity strategies in research, clinical care, education and innovation for radiologists	To provide a practical approach to advancing equity through evidence-based strategies in the four pillars (research, clinical care, education, innovation)	Service delivery—radiology	An overview without defined methodology. For each of the four pillars, an overview of existing barriers and gaps, and of current best practices, is presented with examples, based on the literature	In research, there are under-represented communities such as rural and native populations. Regarding clinical care, disparities in access result from a variety of factors including medical mistrust, varying familiarity with healthcare systems, implicit bias by practitioners or patients, and race-based algorithms. Medical students with increased education on the social determinants of health are more confident when working with underserved populations. Regarding innovations, there is increasing use of AI in radiology but there are potential biases because of incomplete data.	The recommendations are: efforts to recruit underrepresented groups as research participants; Patient navigator programs and community health workers to mitigate barriers to care; More emphasis on social determinants of health in radiology education and recruitment of radiologists among under-represented groups; and Enhancing equitable uptake of emerging radiology innovations.
Asnaani (66) What role can (and should) clinical science play in promoting mental health care equity?	1) To provide a summary of the documented mental health care inequities; 2) To briefly review recent movements to address these inequities, such as social justice and equity	Mental health equity	Review without description of the method	Several areas of scholarship were reviewed in terms of their contributions to promoting mental health care equity, namely: community-based research and community-driven mental health treatment adaptations; task-shifting efforts in domestic and global settings; utilization of technology innovations to promote such work and increase access; and policy efforts. Several ongoing structural inequalities related to social determinants of health were identified as underlying causes of inequitable mental health care, including language barriers, lower financial resources for many historically minoritized groups, immigration complications, and the experience of ongoing racism and discrimination within the health care system. Other persisting barriers to mental health care include stigma toward mental health, a scarcity of treatments that have been tested and validated in minoritized identity groups, and a shortage of culturally responsive treatment providers	It is recommended to examine the effectiveness of culturally adapted and culturally driven interventions, and to investigate why or how such community-based interventions are successful, with an interdisciplinary lens. Open science approaches are encouraged across clinical science to improve adherence to core principles of the discipline, including transparency (with data sharing), ethics, and replicability/reproducibility, all of which are relevant to the study of diverse societies. It would serve the field (and our society overall) well to adopt a stance that diversity science truly applies to all psychological science. Clinical psychologists should take on the challenge/responsibility to incorporate the principles reviewed in this article that are central to promoting mental health care equity across all segments of society, across psychological phenomena, and across professional roles.
Washington et al. (61) A systematic review of the effectiveness of cervical cancer screening and prevention interventions for African American women: implications for promoting health equity	(1) To describe the characteristics of screening and prevention interventions that target African American women; (2) To compare the effectiveness of these interventions; and (3) To determine whether these studies address health equity factors.	Prevention: cervical cancer screening	A systematic review and meta-analysis registered with PROSPERO and guided by the PRISMA guidelines. Reference sections of included studies and relevant systematic reviews were also searched for additional articles. Study	23 articles met inclusion Criteria. There was a wide variety of intervention strategies: community health workers, patient navigation, patient reminders, self-sampling collection, and Human papilloma virus (HPV) vaccination. Cultural tailoring and community-based methods were commonly used, with several studies showing increased screening behavior and knowledge.	This review supports the importance of incorporating health equity principles and community-based methods in screening and prevention interventions. Future research and practice should incorporate African American women's perspectives in intervention development and implementation. Two major implications for future research and practice are self-sampling and deep cultural tailoring.
			quality was assessed. The review also used the Healing ARC framework and Ford's Public Health Critical Race (PHCR) praxis (81) for additional health equity assessments.	Meta-analysis showed that interventions significantly increased the likelihood of participating in cervical cancer screening (OR: 2.43, 95% CI: 1.47–4.02). Health equity assessment revealed that approximately half of the studies struggled to address health equity concerns, while others incorporated cultural tailoring or community-based methods effectively. Few studies acknowledged the impact of racism and structural inequities explicitly.	
Cykert (58) A path toward health care equity: system- based interventions for change	1) To review recent studies that used system-based interventions to reduce disparities and improve outcomes for everyone in North Carolina; 2) To outline how clinicians can apply results to practice.	Service delivery—chronic disease	An overview without defined methodology. Encompasses successful systemic strategies to reduce disparities in chronic disease treatment and outcome.	Communities of color, in particular Black people, have worse health outcomes than white people, in cancer, chronic disease, maternal health and infant mortality. This is due to social determinants of health, (SDOH) but also other factors such as poor patient-clinician communication, mistrust, or clinician implicit bias. According to outcomes, the strategy based on principles of real-time transparency, accountability and enhanced communication was successful in patients with cancer, diabetes and hypertension.	Community insights into barriers and solutions are imperative for building systemic solutions. The principles that reduced disparities and improved health care in chronic diseases could also apply to maternal and child health: Transparency through real-time digital data; accountability through quality improvement that is mindful of disadvantaged groups; and serial enhanced communication incorporating community voices. To truly achieve health equity, additional efforts on SDOH—such as access to health insurance, healthy foods, and a living wage—coupled with interventions to attenuate the physiologic effects of experienced racism, will be needed.
Bell et al. (77) Can evidence drive health equity in the COVID-19 pandemic and beyond?	1) To systematically search, identify, and collate published, well-described, and policy-relevant approaches in which someone has applied epidemiological methods to COVID-19 pandemic inequities in healthcare and health outcomes; 2) To critically assess the potential of proposals for addressing pandemic-related health inequities.	Service delivery—COVID-19	A scoping review following the PRISMA-ScR guidelines. Eleven databases were searched for relevant articles published from January 1st 2020 through February 17 2021 to synthesize published scientific literature describing policy-relevant and evidence-based approaches using epidemiological methods to address health inequities related to the COVID-19 pandemic.	77 papers were included. Significant health inequities affected infection rates, morbidity, and mortality among different socio-economic and racial groups: Inequitable access to testing and vaccines in marginalized communities; disparities in treatment access particularly for culturally and linguistically diverse groups; exacerbation of existing non-COVID-19 health issues due to disruptions in healthcare services and social determinants of health; and other inequities such as race, socioeconomic status, and gender. Proposed solutions target: the inequity in risk of infection, morbidity, and mortality from COVID-19; the inequity in access to testing and vaccines for COVID-19; the inequity in access to treatment for COVID-19; multiple inequities in COVID-19; and non-COVID-19 morbidity and mortality. Some of the proposed solutions, however, could unintentionally exacerbate health inequities .	Health policymakers should co-create, co-design, and co-produce equity-focused, evidence-based interventions with communities, focusing on those most at risk to protect the population as a whole. They should target structural systems of disadvantage which place entire communities at increased risk. Policymakers and practitioners need to examine algorithms for potential discrimination from in-built biases in the data or decisions made in their development. Epidemiologists collaborating with people from other relevant disciplines may provide methodological expertise for these processes. There is a need for robust, evidence-based interventions to combat systemic health and social inequities to allow everyone in our communities to thrive.
Boden-Albala et al. (67) Use of community-engaged research approaches in clinical interventions for neurologic disorders in the United States a scoping review and future directions for improving health equity research (67)	1) To identify and synthesize the intervention studies that have actively engaged with the community in the conceptualization and implementation of interventions to reduce disparities in	Community engagement inr	A scoping review following PRISMA guidelines and Arksey and O'Malley's 5-stage model: (1) Identify the research question; (2) Identify relevant studies using a systematic search strategy; (3) Select studies;	53 studies were included. Community engagement strategies were integrated into interventions in various forms with some studies using multiple approaches. Local organizations as community partners were used by 42% of the studies. Culturally Tailored Materials and Mobile Health (mHealth) were used in 40% of studies to	It is recommended: to involve the community early and continuously, aligning objectives and expectations with the community through a collaborative process; To build curricula that address challenges to community engagement; To prioritize Inclusion of community engagement reporting in peer-reviewed journals; To prioritize and incentivize research that will
	neurologic conditions; 2) To describe the common community engagement processes used by the studies.		(4) Chart (synthesize, code, and interpret) the data; and (5) Collate, summarize, and report the results. The articles were screened and reviewed using Covidence. The review focused on neurologic conditions such as stroke, Alzheimer disease and related dementia, epilepsy, Parkinson disease, spinal cord injury, and traumatic brain injury.	improve accessibility and health promotion. An example is Boden-Albala's trial, which reduced systolic blood pressure in Hispanic stroke survivors. Community health workers were employed by 32% of the studies, enhancing trust and effective delivery of interventions. Faith-based organizations and local businesses were involved in 28% of interventions. Focus groups/health need assessments were a strategy for 25% of studies. Community Advisory Boards were utilized in 19% of studies for feedback and feasibility. Personnel was recruited from the community/champions in 19% of studies. Finally, caregiver/social support was a strategy used in 15% of studies.	identify best practices around community engagement to enhance our understanding of subpopulations who experience disparities.
Gréaux et al. (71) Health equity for persons with disabilities: a global scoping review on barriers and interventions in healthcare services	1) To provide a comprehensive global overview of access to healthcare services for persons with disabilities—the barriers they face and the interventions to remove these barriers; 2) To provide insights to inform the actions that governments and other key stakeholders can take to respond more efficiently to the requirements of the United Nations Convention on the Rights of Persons with Disabilities (UNCRPD).	Access to healthcare—persons with disabilities	A scoping review following the methodological framework proposed by Arksey and O'Malley (see previous review summary). Scholarly databases and the websites of UNCRPD, and reviewed evidence shared during WHO-led consultations on the topic of health equity for persons with disabilities. One hundred and eighty-two articles (published between 2011 and 2022) were included in the review.	182 articles (published between 2011 and 2022) were included. The majority originated from high-income countries. Barriers were identified worldwide across different levels of the health system and through wider contributing factors of health inequities that expand beyond the health system. Human resources issues, the lack of reliable disability data in healthcare services, financial issues, lack of leadership and policy alignment, availability and quality of services, lack of accessible or specialized medical and rehabilitation equipment, products, and devices, the lack	Health needs and priorities of different groups of persons with disabilities can differ widely and require tailored actions. Addressing the barriers faced by the most marginalized groups of persons with disabilities can foster health equity for everyone. Service providers, policymakers, and stakeholders should consult with persons with disabilities with a wide range of intersectional identities to better understand and address their unique health needs and intersectional mediating and risk factors to improve access to healthcare services.
				of disability guidelines and legislation enforcement, and the negative attitudes toward persons with disabilities across all strata of society were major barriers. Socio-cultural discriminatory beliefs about disability and Internalized stigma by persons with disabilities could also impact their access to healthcare services. SDOH factors, lack of opportunities for developing health literacy and limited availability of accessible transport are other problems. Eighteen interventions targeting the negative societal attitudes toward persons with disabilities were identified. However, the interventions to promote equitable access to healthcare services for persons with disabilities were not readily mapped onto the needs, their sources of funding and projected sustainability were often unclear, and few offered targeted approaches to address issues faced by marginalized groups of persons with disabilities with intersectional identities.	Special considerations should be given to the needs of women and girls, sexual and gender minority groups, children and older persons, ethnic minorities, and immigrants and refugees with disabilities. Governments and decision-makers in the health sector should be encouraged to set expectations and establish a collaboration mechanism to work efficiently with Organizations of Persons with Disabilities. Global health decision-makers and funders, in close collaboration with Organizations of Persons with Disabilities have a key role to play in overseeing and coordinating the distribution of resources, building the capacity of country partners, prioritizing the most disadvantaged, and monitoring progress on health equity for persons with disabilities worldwide. A global research agenda is needed, and its development requires the close collaboration and engagement of multisectoral partners and research networks to better address the deep and multidimensional roots of health inequities.
Meadows et al. (63) Strategies to promote maternal health equity: the role of perinatal quality collaboratives	1) To examine the role and strategies of perinatal quality initiatives and collaboratives to deliver safe and equitable	Maternal health equity	A narrative review exploring the contribution of perinatal quality improvement (QI) projects	Perinatal quality improvement is a method to increase obstetric safety and promote health equity. The authors identified six equity-promoting QI strategies, provided	It is recommended: to establish and maintain a culture of equity within healthcare systems; To use data to identify gaps in care and track progress over time; To engage and
	maternity care; 2) To show the evidence of demonstrated success.		in hospitals, health systems, public health departments, or state perinatal quality collaboratives to address equity in maternal outcomes.	examples and characterized each using a classification system based on Bingham's ABCDE'S of QI Strategies and Tactics A: Accountability; B: Buy-in (incentives or disincentives); C: Collaboration and Communication; D: data; E: education; and S: structure change.	collaborate with a diverse set of strategic partners and stakeholders; To include patients and communities in the design and implementation of QI interventions; To educate clinicians on evidence-based practices and the impact of bias and racism on maternal health outcomes; To implement standardized protocols and safety bundles to minimize variations in practice and improve care quality. In sum, leaders should prioritize maternal equity, acknowledge racism's impact on health outcomes, and invest in staff education and data systems to improve care quality and equity.
Peek et al. (78) Advancing health equity through social care interventions	1) To use evidence on addressing racism in social care intervention research to create a framework for advancing health equity for all populations with marginalized social identities; 2) To recommend how the Agency for Healthcare Research and Quality (AHRQ) could advance health equity for marginalized populations through social care research and care delivery.	Social care interventions	Overview without defined methodology. This commentary is informed by a literature review of social care interventions that were affiliated with healthcare systems, input from health equity researchers, policymakers, and community leaders attending the AHRQ Health Equity Summit; and consensus of the authors.	Groups with marginalized social identities have disproportionate social needs (e.g., food insecurity) and negative SDOH. Funders and healthcare systems are interested in addressing patient's social needs and community-level social determinants of health as part of comprehensive healthcare strategies to reduce health inequities. However, few social care intervention studies have conceptualized race as a proxy for exposure to racism or examined differential treatment effects of the intervention by race or ethnicity. Addressing specific sociocultural priorities of populations with marginalized social identities is an important strategy to increase the effectiveness of social care interventions.	The authors recommend that AHRQ: (1) Create an ecosystem that values research on SDOH and the effectiveness and implementation of social care interventions in the healthcare sector; (2) Work with other federal agencies to (a) develop position statements with actionable recommendations about racism and other systems that perpetuate marginalization based on social identity and (b) develop aligned, complementary approaches to research and care delivery that address social marginalization; (3) Advance both inclusive care delivery and inclusive research teams; (4) Advance understanding of racism as a social determinant of health and effective strategies to mitigate its adverse impact on health; (5) Advance the creation and scaling of effective strategies for addressing SDOH in healthcare systems, particularly in co-creation with community partners; and
					(6) Require social care intervention researchers to use methods that advance our understanding of social health equity.
Ukke et al. (59) Lifestyle Interventions to prevent type 2 diabetes in women with a history of gestational diabetes: a systematic review and meta-analysis through the lens of health equity	To assess the prevention of type 2 diabetes (T2DM) in women with prior gestational diabetes (GDM) using population characteristics according to the PROGRESS criteria: place of residence, race/ethnicity/culture/language, occupation, gender/sex, religion, education, socioeconomic status, and social capital	Behavior change	Systematic review and meta-analysis. Databases were searched for interventional studies of diet, physical activity, or behavioral interventions published up to February 2023. Random effects subgroup meta-analysis was conducted to evaluate the association of population characteristics and intervention effects. Randomized controlled trials, non-randomized controlled trials, and pre- post-single-arm studies were included. Were excluded studies combining pharmacological or supplementation components with lifestyle intervention.	40 unique studies were included. The meta-analysis included 26 studies that reported the primary outcomes using the PROGRESS criteria. Two-thirds of the studies reported on race/ethnicity and education level. Less than one-third reported on place (urban/rural), occupation, and socioeconomic status. None reported on religion or social capital. Lifestyle interventions from high-income countries showed a greater reduction in bodyweight, a key factor for the prevention of T2DM, compared with the studies conducted in middle-income countries for subgroup difference. In the studies that did report findings based on the PROGRESS criteria, participants were mostly (73.9%) tertiary educated and had a high level of income (61.5%). This review highlights the lack of inclusion of participants at highest risk of T2DM. There were no studies in low-income countries in Africa or the Pacific region despite these regions being being disproportionately burdened with T2DM and GDM. Virtually delivered interventions have a better effect than those with both virtual and in-person components, with in-person delivered interventions being the least effective in women with a history of GDM.	Substantial heterogeneity between studies needs to be considered when interpreting the results of this meta-analysis. The high risk of bias in most studies needs to be considered when applying the results of this meta-analysis. There are ethnic disparities in the overall prevalence of T2DM as well as in the progression of GDM io T2DM, and an adequate representation of ethnic groups bearing the greater burden of the disease and the disaggregation of data, where feasible, is needed in the research to better understand the effectiveness of interventions in these groups. To advance the understanding of T2DM prevention in all population subgroups, future researchers and funders need to close the equity research gap in the prevention of T2DM in women with a history of GDM by focusing on the inclusion of disadvantaged groups (or groups which are under-represented) and by collecting and reporting disaggregated data on equity.
Kanengoni-Nyatara et al. (73) Barriers to and recommendations for equitable access to healthcare for migrants and refugees in Aotearoa, New Zealand: an integrative review (73)	To synthesize the evidence on barriers to accessing healthcare services and where present, propose interventions to improve services in various healthcare settings for migrants and refugees	Access to healthcare -migrants and refugees	An integrative review following the PRISMA guidelines. Included studies published between January 2016 and September 2022 to mirror the adoption of the 2030 Sustainable Development Goals in 2015. Data were thematically analyzed using vote counting to identify frequent themes, which were refined through discussion. A narrative synthesis was then used to integrate the findings and highlight relationships among the themes.	Out of 237 identified studies on migrants and refugees, 13 were included in the review. All except one were qualitative, and the other one used a mixed methodology. Most studies focused on refugees. Participants were from LMICs or non-English speaking countries. Studies predominantly involved women. Attitudinal barriers included the lack of culturally competent healthcare providers, discrimination by healthcare providers, and personal, social, and cultural attributes. Structural barriers referred to policies and frameworks that regulated the accessibility of health services such as the cost of healthcare, accessibility and acceptability of interpreter services, length of allocated appointments and long waiting times for an appointment, difficulties navigating the health system, and logistical barriers. Mobility barriers were also reported during COVID-19 lockdowns where participants' support services were disrupted. For mothers who could not drive or did not have a car, using public transport to access healthcare was particularly difficult for those who had two or more children	The authors recommended: 1) Fostering a Sense of Belonging: people from former refugee backgrounds to influence policy makers to recognize the unique individual, social, cultural and historical factors that affect their health and promote a culture of acceptance that celebrates diversity; 2) Enabling a Whole-of-Society Approach, with collaboration between healthcare providers and non-governmental organizations, the integration of a gender perspective, and community engagement; 3) Government, Organizational Structures, and Policies: Implementation of culturally centered policies, funding for interpreter services, addressing structural barriers, and improving healthcare workforce diversity.
Jackson-Triche et al. (68) Achieving mental health equity: collaborative care	To review what is known about the impact of integrated care programs on improving	Collaborative care (CC) in mental health/behavioral health	Rapid literature review of reviews and individual studies. No detail on methodology.	The review gives strong evidence that CC is a model that has the potential to reduce disparities for ethnic minorities and other	To fully realize the promise of CC, there is a need for approaches that focus on effective community engagement, coalition building,
	health equity, with special emphasis on collaborative care (CC)			at-risk populations who are often poorly served by usual primary care systems, and who have lower engagement and health outcomes because of other underlying risk factors. As a systems-based approach, CC has been shown to not only improve access to care but also to improve the quality of care received and health outcomes.	and cultural adaptation, as well as developing innovative approaches such as addressing social determinants. The authors argue that key first steps are using health equity-focused strategies when planning and implementing CC and giving careful thought and attention to engaging diverse populations and considering their specific preferences and needs.
Kibakaya and Oyeku (65) Cultural humility: a critical step in achieving health equity	To identify and discuss research pertaining to cultural sensitivity in pediatric primary care and comment on its role for achieving health equity.	Cultural sensitivity	Overview without defined methodology. Culturally sensitive care is defined as “the delivery of care within the context of appropriate physician knowledge, understanding, and appreciation of cultural distinctions.” An alternative concept is one of cultural humility, which incorporates elements of self-questioning, immersion into an individual patient's point of view, active listening and flexibility, which all serve to confront and address personal and cultural biases or assumptions.	There is scarcity in breadth and depth of existing literature that addresses culturally sensitive interventions in pediatric primary care. There is a notable deficiency of research tackling the array of medical, developmental, social, and emotional issues that primary care providers address daily. One current strategy that health systems and educational institutions are leveraging to reduce health disparities is addressing the role of implicit bias and structural racism. Medical schools in the USA have developed various curricula incorporating elements of cultural competence.	Demonstrating cultural humility frees health care professionals from having to possess expert knowledge about a myriad of cultural differences and fosters open communication with patients to achieve shared health and developmental outcomes. The authors argue that it is imperative that healthcare professionals work in partnership with patients and their families to reduce health disparities. The authors and other researchers posit that cultural sensitivity may improve physician-patient communication and collaboration, increase patient satisfaction, and potentially enhance treatment adherence, improve clinical outcomes and reduce health disparities.
Doyle et al. (57) Achieving health equity in hypertension management through	To examine recent literature on the social determinants of health as they relate to	Hypertension management	Overview without defined methodology. Examples from the literature of the	Positive impact on behavior or outcomes was shown in minorities by improving access to resources, behavior counseling, education,	There is a need for innovative methods to modify the factors that affect health upstream (and that can be modified, at variance with
addressing the social determinants of health	hypertension and cardiovascular disease and discuss relevance to the practice of emergency medicine		intersection of social determinants of health and hypertension management or outcomes are given.	the action of community health workers, and technology.	race and ethnicity) before the symptoms appear, such as education and neighborhood characteristics. Multidimensional partnerships involving healthcare systems, communities, public health organizations and social welfare entities are important to better prevent and manage hypertension through action on social determinants of health.
Richard et al. (70) Equity of access to primary healthcare for vulnerable populations: the IMPACT international online survey of innovations (70)	To identify, refine and then trial best practice innovations to improve access to PHC, particularly for vulnerable populations	Access to primary health care	As part of IMPACT project, an overview (environmental scan) of the breadth of current innovations from the field. The authors distributed a brief online survey to an international audience of PHC researchers, practitioners, policy makers and stakeholders using a combined email and social media approach. Respondents were invited to describe a program, service, approach or model of care that they considered innovative in helping vulnerable populations to get access to PHC. Written descriptions of innovations were mapped against the framework of Levesque et al. (74) to identify which access dimensions were involved.	The study collected 240 unique examples of innovations, which were primarily health sector focused (71.3 %). Almost all innovations were operating at the practice or community level (90.4 %). Most innovations addressed supply-side dimensions of access, with less focus on demand-side dimensions. Few innovations targeted both supply- and demand-side dimensions simultaneously. The study also noted that many innovations were funded by government sources and were primarily implemented in community health settings.	Increasing efforts are needed to address both supply- and demand-side dimensions of access simultaneously to improving the effectiveness of innovations. More comprehensive and integrated approaches are needed to achieve transformative change in access to PHC for vulnerable populations. More research is needed, in particular for more rigorously undertaken systematic evaluations of initiatives that are developed, considering the context in which innovations are implemented and having indicators which cover the broad range of access determinants (health and social) for accurate measurement of the effects of intervention components on specific access dimensions.
Schneider et al. (55) Increasing equity while improving the quality of care	To highlight the efforts to reduce inequities in the quality of cardiovascular care, building on insights from recent scholarship on the effects of structural racism in the broader society and also within medicine.	Cardio-vascular care	Overview without defined methodology. The authors adapt a tool frequently used in quality improvement work—the driver diagram, which maps out a path toward an intended outcome—to chart likely areas for diagnosing root causes of disparities and developing and testing interventions.	There are persistent racial and ethnic disparities in cardiovascular disease (CVD) outcomes, particularly among Black, Latino, Asian, Pacific Islander, and Indigenous populations. In the case of heart failure mortality, disparities have widened over time. Clinical and behavioral risk factors like hypertension, diabetes, diet, and tobacco use partly explain these disparities. Genetic factors play a minimal role. There are persistent disparities in care quality among racial and ethnic groups. The authors developed an equity-centered quality improvement model and a roadmap to advance cardiovascular health equity as guides to improve the measurement and analysis of quality problems and the implementation of care interventions and policies that reduce racial and ethnic disparities in outcomes.	The Equity-Centered Quality Improvement Model explicitly maps the many influences within and outside of health care that contribute to inequitable patient outcomes. Reducing discriminatory interactions with patients and families and enhancing access to care can increase the trustworthiness of institutions and professionals. Physicians, other health professionals, and health care systems can reduce racial and ethnic disparities in cardiovascular mortality and other outcomes if they simultaneously and intentionally address both quality and equity. Designing interventions should take a broader perspective than modifying care for patients while they are in a clinical care setting. Tailoring solutions to patients and their communities may involve actively engaging patients and community health workers in developing and evaluating interventions. The use of geospatial and clinical data is recommended to identify disparities, diagnose their root causes, and design targeted interventions. Engaging patients and community health workers in developing and evaluating culturally tailored interventions is also needed.
Kohler et al. (36) Population-based physical activity promotion with a focus on health equity: a review of reviews	To identify current evidence on the effectiveness of population- based physical activity (PA) promotion in the community with a particular focus on health equity.	Prevention—physical activity promotion	Umbrella review of systematic reviews on population- based PA promotion for the period 2015 to 2021. Six electronic databases were examined. A reference list and gray literature search were also conducted. A quality assessment was conducted for each identified review. All included reviews of population-based approaches for PA promotion with a focus on disadvantaged populations.	Six reviews were included, and they were all rated as high quality. Mass-media campaigns, point-of-decision prompts, environmental approaches, policy approaches, and community-based multi-component approaches can promote PA in the general population. Across populations with social disadvantages, mass-media campaigns, point-of-decision prompts and policy approaches are likely to be effective if they are tailored. However, none of the reviews on community-based multi-component approaches provided evidence on health equity.	Future studies should assess the theoretical basis of these approaches, their differential impact including the potential negative and unintended consequences as well as the long-term impact on PA promotion and health equity. Tailoring interventions to the needs of disadvantaged populations, and engaging people with social disadvantages in the development, implementation and evaluation of population-based PA programs for their empowerment are recommended, as well as community-based multi-component approaches combining structural (environment and policy) and behavioral components.
Arsenault et al. (64) Equity in antenatal care quality: an analysis of 91 national household surveys	To identify the reasons for inequitable access of women for antenatal care in low- and middle-income countries (LMICs).	Antenatal care based on economic status of the country	A systematic review of antenatal care quality using information from 2007 to 2016 Demographic and Health Survey (DHS) and Multiple Indicator cluster surveys (MICS).	The study shows that there are much lower and inequitable levels of quality in many LMICs even though they reached high levels of antenatal care coverage. The wealthiest women were four times more likely to report good quality antenatal care than the poorest. Poorer people mostly live in rural areas where there are poorly functioning health systems. Other factors that influence antennal care inequalities include availability of good facilities nearby, cost of diagnostic procedures, provider discrimination or bias, and incitement of a patient to seek high-quality care, skills of care providers and equipment available.	Equity in effective coverage should be used as the new metric to monitor progress toward universal health coverage. However, assessing the social inequalities for different nations is still a challenge. The study suggests that more work is still needed to understand factors responsible for inequities in health-care quality. The article recommends better measurement and systematic improvement in healthcare quality especially in poor and vulnerable populations. In the Sustainable Development Goal era, achieving parity in health outcomes between rich people and poor people, within and across countries, will require greater focus on the quality of health services and its equitable distribution.
Davy et al. (72) Access to primary healthcare services for indigenous peoples: a framework synthesis	1) To identify issues that hinder Indigenous peoples from accessing primary health care; 2) To explore how these were addressed by Indigenous health care services.	Access to primary healthcare—indigenous peoples	A thematic review of 50 studies with focus on access to primary healthcare services for indigenous peoples using Levesque's accessibility framework (74). These studies were from the United States, Canada, New Zealand, South America, and Papua New Guinea.	Issues relating to the cultural and social determinants of health (such as unemployment, poverty, and low levels of education) influence whether Indigenous patients, their families and communities were able to to access health care. Indigenous health care services addressed these issues in several ways the provision of transport to and from appointments, a reduction in health care costs for low-income people and close consultation with community members in identifying and then addressing health care needs.	Indigenous health care services appear to be best placed to overcome both the social and cultural determinants of health which hinder Indigenous peoples from accessing health care. Common factors for successful navigation include the importance of culturally safe and wherever possible, locally owned Indigenous health care services. Findings also suggest that Levesque et al.'s accessibility framework (74) should be broadened to include factors related to the health care system, such as funding. Mainstream services are set up to cater to dominant, often non-Indigenous cultures with a set of socially constructed values and norms which can be at odds with Indigenous communities' beliefs and values. The authors acknowledge a need to further explore factors relating to the health care system which facilitate or impede access to Indigenous health care services.
Tao et al. (75) The impact of reimbursement systems on equity in access and quality of primary care: a systematic literature review	To compare the different types of reimbursement system in relation to socioeconomic and racial inequalities in access, utilization and quality of healthcare.	Access to, and quality of primary healthcare	Systematic literature review. Only studies from high-income countries were included. Schemes considered were fee-for-service, capitation, and pay-for-performance (the Quality and Outcome Framework). Seven studies compared capitation and fee-for-service. Access, uptake and quality of services, and chronic disease management were considered.	22 papers on experimental or observational studies conducted in the primary care settings were included. Little scientific evidence supports an association between reimbursement system and socioeconomic or racial inequity in access, utilization, and quality of primary care.	The reimbursement scheme may have a differential impact depending on the outcome under study, the context including various healthcare systems, and social stratifiers other than race or socioeconomic status. A combination of reimbursement schemes, which was observed, does not allow to isolate the impact of a given scheme. Policies for resource allocation that matches the increased healthcare needs of underserved groups might have a greater impact on health inequalities that the type of reimbursement. Further empirical studies are necessary and recommended.
Malou et al. (69) Promotion de la santé globale et approche socio-écologique de l'autodétermination chez les personnes présentant une déficience intellectuelle: une revue systématique des interventions|	To analyze the contexts and highlight conclusive results of interventions that promote overall health and the place of self-determination among people with intellectual disabilities (ID).	Behavior change	Systematic review of interventions promoting self-determination and overall health among people with intellectual disabilities. The paper analyzed relevant interventions focusing on individuals with ID, on environment, and on both ID and environment.	Significant positive results of both types of interventions were observed from a quantitative and qualitative point of view when different factors such as the interaction between individuals and the environment in a broad sense were considered (material, human, living environment, etc., development of self-determination and improving health literacy).	Future actions should further evaluate the improvement of overall health through the development or strengthening of self-determination both at the individual and environmental level.
Gandhi (76) Charting the evolution of approaches employed by the Global Alliance for Vaccines and Immunizations (GAVI) to address inequities in access to immunization: a systematic qualitative review of GAVI policies, strategies and resource allocation mechanisms through an equity lens (1999–2014)	To assess how GAVI's approach to address equity/inequity in immunization has evolved over time	Access to immunization	A systematic qualitative review on the evolution of GAVI's focus on reducing inequities in access to vaccines, immunization, and GAVI funds allocated between and within countries. The review included electronic databases search and a direct review of available GAVI Board papers, policies, and program guidelines.	Over time, GAVI has progressively added vaccines to its portfolio. This expansion should have addressed inter-country, inter-regional, intergenerational and gender inequities in disease burden. However, evidence is scant with respect to final outcomes. A focus on addressing inequities between higher-income countries and lower-income countries may reflect the viewpoint of the Alliance stakeholders. In terms of resource allocation mechanics and program policies, GAVI focused almost exclusively on between-country equity concerns.	By building on its successes, GAVI is well-positioned to bring the benefits of vaccination to previously unreached and underserved communities toward provision of universal health coverage. Reliance on national averages makes sense when speaking of vaccines that are generally regarded as highly equitable interventions (e.g. targeting boys and girls alike) and most importantly, capable of conferring population-wide herd immunity benefits against vaccine-preventable diseases at high enough levels of coverage. Future research should illustrate the evolution and quantitative effects of GAVI's efforts to address between- and within-country inequities in access to new vaccines, utilization of immunization services, access to GAVI resources, and impact on vaccines preventable diseases.
Nelson et al. (60) Achieving health equity in preventive services: a systematic review for a National Institutes of Health pathways to prevention workshop	1) To examine the effects of barriers that create health disparities in recommended preventive services for adults; 2) To evaluate effectiveness of interventions to reduce them.	Preventive services	Systematic literature review of preventive services related to cancer, cardiovascular diseases, and diabetes in adults (1996–2019).	120 studies were synthesized. Disadvantaged populations in the USA experience disparities in the use of preventive health services. Patient navigation services increased colorectal, breast, and cervical cancer screening rates. Some patient navigation interventions included additional services such as reminder calls, lay health workers, etc. Telephone calls and prompts improved colorectal cancer screening. Reminders from lay health workers improves breast cancer screening.	Further research is needed to address gaps and deficiencies of existing studies, and involving unstudied populations experiencing adverse effects of healthcare disparities: racial and ethnic minorities, socioeconomically disadvantaged populations, underserved rural populations, sexual and gender minority populations, and others subject to discrimination.

## 3 Results

This scoping review of reviews identified 50 reviews including four reviews of reviews and 12 overviews. It showed that predominant actions taken to foster health equity were centered around health service delivery (26/62): primary healthcare, as well as specialized medical care, preventive services, maternal care, access to vaccines specifically for COVID-19, and mental health. Marginalized or underserved communities such as indigenous people, refugees and immigrants, ethnic minorities, and persons with disabilities were targeted in several reviews. Thirteen reviews (13/62) addressed health equity through governance and policy, including practice guidelines, policy reforms, addressing the social determinants of health (SDOH), and organization capacity assessment. The reviews on information and evidence data (7/62) focused on methods to assess and report the impact of interventions on health equity. Two reviews addressed the effects of climate change and CO_2_ reduction actions on health inequities; these were the only ones that considered the ecosystems beyond human health. Reviews on technologies (11/62) pertained to digital health equity, healthcare algorithms, and the role of Artificial Intelligence (AI). The reviews on human resources and equity (5/62) referred to the training of nurses, other health professionals, and community health workers, as well as community participation. Details on the reviews are provided in Table 1. Of note, the same type and amount of data cannot be provided for all reviews, as the information available varied considerably across the papers and furthermore, since the quality of the reviews was not assessed. Notwithstanding, the focus was on the findings. Following the table, an overview of the reviews by key area is provided.

### 3.1 Governance and policy action

Out of the 13 reviews about governance and policy approaches for health equity, three were outside the health sector. One reviewed the importance of neighborhood policies in the USA to advance health equity through institutions, the physical, and the social environment (17). The recommended measures included fair housing laws and strengthening of community-led initiatives to improve material and social conditions. The second one reviewed promising strategies to advance addressing root causes of health inequities, including the analysis of structural power structures to address equity issues (18). The third one reviewed policies that perpetuate health disparities in children in the USA (19). Several social and structural determinants of health were considered, such as housing highlighted in one review (17), the criminal legal system, and immigration. Supporting community organizations, in this case those that support immigrant communities, is recommended. Which social determinants of health the healthcare systems have the capacity to address is an interesting question raised in two review papers on health sector actions (20, 21).

Reviews on health sector actions (*N* = 10) pertained to clinical practice guidelines and their development (22, 23), and to various strategies for healthcare systems (20, 21, 24–26).

Regarding clinical guidelines, health equity promoting practices need to be integrated from the onset of their development, and community organizations must be among the involved stakeholders. Healthcare system strategies include health equity as part of health system performance assessment (20) and organizational capacity for health equity assessment (25) or strengthening (24). The results of a primary health care (PHC) policy reform in Sweden were assessed, including the impact on health equity (27). It was found that while the reform increased access to PHC and the number of visits, the improvements were primarily in affluent areas and among people with lower health needs. The study showed that resources were more influenced by provider location, patient choice and demand rather than need, which suggested potential damage to health equity. The effect of decentralization on health equity was assessed in a systematic review encompassing a quality assessment of the studies (26). The results were mixed, with a risk of increased disparities due to financing; central coordination and redistribution were deemed necessary. One review on neonatal health disparities emphasized the quality of care (28). Another review (29) assessed the implementation of guidance frameworks provided to organizations implementing interventions to make care and outcomes more equitable by changing policies and practices. Several models and frameworks were analyzed, and most of them concentrated on the organization's external context, such as analysis of data on race and ethnicity. Also, addressing the inner context such as readiness for change was deemed important. As part of health system strengthening approaches for health equity, one review (24) concluded on the need for broadened frameworks to measure intersecting forms of social disadvantage. Among the strategies for healthcare systems in the USA to address social determinants of health and improve health equity in the pediatric population, the value-based payment is described as promising (21). This system has the goal of supporting pediatricians in intervening on upstream influences on health to reduce long-term cost. Incentives are used to address the social determinants of health through universal screenings, referrals to community-based organizations, and investing in various supports.

### 3.2 Information and evidence data

Seven reviews were classified under this theme. In a systematic scan of 18 USA-focused clearinghouses that assigned an intervention effectiveness rating for improving community health and the social determinants of health (30), it was found that less than half provided information on the potential impact on health equity. These clearinghouses defined and operationalized health equity in different manners. They lacked transparency in their methods and used various approaches to communicate the findings. Clear and transparent definitions, values, and methods would be needed.

Action is guided by conceptual frameworks. One review (31) summarized methods, frameworks, or tools used as a conceptual basis for investigating dimensions of health equity impact in systematic reviews of public health interventions. In this overview of reviews, planned methods for conceptualizing equity impacts were fully applied in less than half of the reviews. The predominant framework was PROGRESS-Plus, used in more than one-third of the reviews. However, there are conceptual and measurement issues owing in part to the lack of standardized definition, operationalization, and measurement of health equity dimensions. Additionally, the nature of the differential impacts is complex, and dimensions of health equity—access to services; quality of those services; and health outcomes—may interact with each other.

WHO has developed several health inequality monitoring resources as part of its 2022–27 inequality monitoring and analysis strategy; these were reviewed (32). The resources include a health inequality data repository, a health equity assessment toolkit (HEAT and HEAT Plus), health inequality monitoring tools and resources including a handbook, step-by-step manuals and statistical codes, and eLearning courses. Health inequality reports focusing on specific health topics or countries are periodically released. The WHO strategy and tools respond to the need for high-quality evidence on health inequalities to advance health equity.

Participatory implementation science may be regarded as a tool or strategy to advance health equity, as shown in an overview with examples from the literature (33). It is an iterative approach that offers an inclusive and collaborative perspective on implementing and sustaining evidence-based interventions to advance health equity. With a focus on health equity, participatory implementation science emphasizes processes for, and impacts of, community engagement, dissemination, social action, capacity building, and systems changes. Systems changes include, for example, assessing power distributions and how they can be shifted to create equity-promoting contexts.

Four reviews provided information or evidence data. One review (34) examined how and whether social needs interventions in multiracial or multiethnic populations in the USA advanced health equity. The interesting framework considered whether the studies were “conceptually thoughtful” in that they helped explain the root causes of racial health inequities and whether they were “analytically informative,” that is, if they examined the effects that differed by race or ethnicity. Out of 152 studies, less than 10% were conceptually thoughtful, and only 14% were analytically informative, and mixed effects on health equity were reported. In a scoping review of publicly funded projects to reduce bias/racism in maternal care in the USA (35), only four publicly funded such interventions were identified since 2018, which reveals an evidence gap. These projects are nonetheless promising as they used innovative strategies, including participatory research responding to community needs, multi-component and multi-level interventions, and human resource training in three out of four cases. A review of six high-quality reviews on community-based promotion of physical activity showed that mass-media campaigns, point-of-decision prompts, and policy approaches could be effective for socially disadvantaged groups provided the messages were tailored (36). However, none of the reviews provided evidence of an impact on health equity. Finally, a review of climate change impact on health inequities and mitigating efforts (37) concluded on the need for a “whole of science” approach to address the current climate change and health inequality crisis as climate change magnifies health inequalities. Cross-cutting initiatives are given as examples of integrative approaches, such as the Pathfinder Initiative, which draws on case studies to improve planetary health; the Pacific Regional Environmental Program; and the Rockefeller Foundation's 100 climate resilient cities' initiative, which develops resilient climate plans. Community-based participatory research, local knowledge, a better understanding of climate inequities, and expanding training opportunities were among promising strategies.

### 3.3 Technologies

Digital health, or telemedicine, is the topic of seven out of the 11 reviews on technologies and health equity. Such technologies may indeed contribute to more equitable access to health services as the digital divide related to consumer information technologies has diminished. A scoping review on barriers and facilitators for digital primary health care in refugees and immigrants found that flexibility of digital modalities was a facilitator while older age and lack of digital literacy were obstacles (38). In another review, the social determinants of health were found to interact to increase or reduce digital equity (39). Three levels of digital divide were identified: lack of access, lack of skills, and lack of possibility to use the tools for one's health. Telehealth for the ambulatory care of various chronic diseases (e.g., heart failure, diabetes, Parkinson's disease, neurological diseases) was usually found to be as effective as in-person care; however, the results on equity among those at higher outcome risk have not been studied (40). In a review on the use of consumer information technologies (CITs) for behavioral interventions in health disparity populations (41), these technologies, including mobile health, telehealth, and social media, showed potential in promoting self-management of chronic diseases, supporting activities like diet and physical activity monitoring, enhancing motivational learning, and providing health education. Such technologies also proved useful for the evaluation of interventions. Another application of digital health is for mental health. In a systematic review, most studies (using direct-to-consumer telehealth videos) observed widening disparities for disadvantaged populations, including rural populations, older patients, and racial/ethnic minorities (42). The generalizability and sustainability of access to digital mental health services for immigrants and refugees was questioned in another review, which showed that participation not only depended on the individual (e.g., literacy), the program (computer and software), and the social context, but also depended on financial and technical support (43). Digital literacy is important for the equitable distribution of e-health resources, as confirmed in a review (44). Screening to identify population groups in need of digital literacy interventions is important to advance health equity.

Carbon capture, utilization and sequestration technologies are promising to reduce greenhouse gas emissions. These have the potential to worsen or improve health equity. The benefits and burdens must be distributed equitably, and the needs and perspectives of the most vulnerable groups must be prioritized (45).

Clinical algorithms are a technology, in a sense, and they may be biased. Strategies to mitigate these biases were reviewed (46). The strategies were technical (e.g., the algorithm development process), operational (e.g., governance), or system-wide (e.g., training on the risk of bias), but no single best practice was identified. How healthcare algorithms impact racial and ethnic disparities was reviewed (47). The algorithms tested referred mainly to kidney function and cardiovascular risk. The evaluation strategies consisted of the removal, addition or modification of variables. The review suggested that mitigation strategies reduced racial and ethnic disparities in care. Artificial intelligence (AI) is another technology that can contribute to health equity. Its impact is still little studied, but a review summarized existing literature on the way AI has the potential to exacerbate or reduce inequities in occupational safety and health (48). AI has the potential to improve occupational safety and health particularly in high-risk industries such as construction and mining. These jobs are mainly filled by workers from racialized ethnic minority groups. AI may also have negative health effects owing to job insecurity, new jobs and income disparities. Social safety nets may improve equity in communities that experience the negative impact of AI integration. Considerable research on both the positive and adverse impacts of AI is direly needed.

### 3.4 Human resources

Only five reviews on health equity focused on human resources, but some reviews on other topics also included training or human resources components. Education has nonetheless emerged as a pivotal factor in promoting health equity. One review is on the integration of nurse practitioners into primary health care as a strategy to deliver comprehensive care and advance equity (49). For their successful integration, six requirements were compiled from the literature at the micro, meso and macro levels, and provided a framework: (1) autonomy and agency; (2) awareness and visibility; (3) shared vision; (4) leadership; (5) funding and infrastructure; and (6) intentional support and self-care. Another review examined nursing curricula to determine to what extent the principles of health equity are explicit in prelicensure curricula (50). Only two papers out of 20 described overall well-integrated explicit health equity content, and there was little attention to whether students transferred this learning into practice. The conclusion was that there is a gap in the health equity content despite the need. A review evaluating over 150 studies carried out in low- and middle-income countries revealed that community health worker programs were effective in reaching the most disadvantaged populations (51). However, such programs achieve better equity in service delivery than outcomes as many individuals still face barriers in adopting health advice and referrals. Another review focused on the teaching of racism as a determinant of health in public health training (52). Few examples of peer-reviewed literature were found on curricula, lessons and competencies developed to better understand racism. The review revealed a lack of consensus on the most effective approach to teaching about racism in public health. Cultural competency and safety as essential for health equity is the object of a review centered on indigenous healthcare in New Zealand (52). The authors highlighted the significance of cultural safety in addressing racism, power imbalances in healthcare, and historical factors affecting healthcare experiences in marginalized populations. Cultural safety training and monitoring within healthcare organizations was deemed essential.

### 3.5 Service delivery

Service delivery includes health equity issues and initiatives in general care, in medical treatment, and in public health interventions. Of a total of 26 reviews, 10 pertained to medical care and three to mental healthcare. Two papers on the care of heart disease reviewed the factors of racial and ethnic disparities from prevention to advanced interventions, and then discussed existing or recommended strategies to reduce these disparities (53, 54). In the USA, the burden of modifiable risk factors for heart disease is higher among Black, Asian, Native, and Hispanic populations. Despite some progress, there are significant gaps in the management of hypertension and diabetes among Black and Hispanic patients because of financial barriers, missed visits, and poor access to prevention. Other factors that drive inequities in treatment include social determinants of health, systemic inequities, and structural racism. Examples of ongoing promising initiatives are the American Heart Association's “Life's Simple 7,” community-level efforts to reduce tobacco consumption and obesity and pay-for-performance programs for more equitable or better-quality programs. More equity in clinical trials, culturally tailored community interventions, cross-cultural training, enhanced diversity in organizations, and recruitment in medicine are among the numerous recommended approaches to improve health equity through the spectrum of care. Another review (55) also highlights cardiovascular outcome disparities, particularly among Black, Latino, Asian, Pacific Islander, and Indigenous populations. Beyond clinical and behavioral risk factors, the clinical management of cardiovascular disease risk factors also shows significant disparities, which extend to the adoption of new technologies. Another review focusing on cardiovascular disease examined how data on social determinants of health were used to improve health equity (56). The social determinants most often considered were healthcare access and quality, followed by the neighborhood and built environment. Few studies focused on economic stability, social and community context, or education access and quality. Data on neighborhoods and built environments were used to determine, for instance, areas with limited access to pharmacies and to draw maps of cardiovascular disease incidence and climate vulnerability. Other authors (57) reviewed the literature on social determinants of health as they related to the management of hypertension and cardiovascular disease. A positive impact on behavior or outcomes was observed in minorities by improving access to resources, behavior counseling, education, the action of community health workers, and technology. In an overview of successful systemic strategies to reduce chronic disease treatment and outcome disparities (58), it was found that according to outcomes, successful strategies were based on principles of transparency through real-time digital data; accountability through quality improvement that is mindful of disadvantaged groups; and serial enhanced communication incorporating community voices. A systematic review focused on the effectiveness of lifestyle interventions for the prevention of diabetes among women who had had gestational diabetes according to social determinants of health (59). The interventions were all carried out in high- or middle-income countries. Studies from high-income countries showed a greater reduction in body weight compared with the studies conducted in middle-income countries. It was noted that a high proportion of participants had higher education or higher income. The conclusion was that the women most at risk may not have been included in the programs. A systematic review (60) explored the barriers contributing to disparities in preventive services and the interventions aimed at reducing these among disadvantaged populations in the USA. It was found that clinician-delivered interventions played a crucial role in smoking cessation while technology-assisted interventions (patient navigations, telephone calls, and community engagement) were linked to positive outcomes, including improved cancer screening rates.

In a review of cervical cancer screening and equity among African American women (61), the meta-analysis showed that interventions significantly increased the likelihood of their participating in cervical cancer screening. A wide variety of intervention strategies were used, that is, community health workers, patient navigation, patient reminders, self-sampling collection, and HPV (human papillomavirus) vaccination.

Evidence-based health equity strategies under the pillars of research, clinical care, education and innovation were reviewed for radiologists (62), but they would also apply to other medical specialties. Regarding research, there are under-represented communities such as rural and native populations. In clinical care, disparities in access result from factors such as medical mistrust, implicit bias by practitioners or patients, and race-based algorithms. Medical students with increased education on the social determinants of health are more confident when working with underserved populations. Regarding innovations, there is increasing use of AI in radiology but there are potential biases because of incomplete data.

A narrative review explored the contribution of perinatal quality collaboratives on perinatal health equity (63). These collaboratives are state-based networks of stakeholders in hospitals, health systems, and public health departments. Their aim is to advance maternal equity through improving the quality of care. All 50 USA states belong to these networks. Six equity-promoting quality improvement strategies were documented in the review and used in the collaboratives (ABCDES): Accountability, Buy-in, Collaboration and communication, Data leverage, Education, and Structural changes. Published papers have highlighted the success of this quality improvement approach to reduce or eliminate racial inequities, based on the occurrence of severe maternal morbidity. A study on the quality of antenatal care in 91 low- and middle-income countries revealed that wealthier women were four times more likely to receive high-quality care compared to poorer women (64). The authors advocated using care quality as a key metric to monitor progress in universal health coverage. Another determinant of health equity is cultural sensitivity. In a viewpoint article on cultural sensitivity in pediatric primary care (65), noted that the literature on this topic is scarce, whether addressing implicit bias or structural racism, although there are some elements of cultural competency included in medical school curricula.

We identified a few reviews on health equity in mental healthcare and care of neurologic conditions (66–68). Marked inequalities in mental health care in minoritized population groups are widely recognized. Among the contributing factors as reviewed (66), social determinants of health are important but other drivers are involved, such as discrimination and racism, stigma regarding mental health, and the lack of treatments tested and validated in minoritized groups. Community-based research and community-driven mental health service adaptations are among the strategies identified, as well as task-shifting efforts in domestic and global settings, utilization of technology innovations to promote such work and increase access, and policy efforts. A scoping review (67) identified various community engagement strategies in the management of conditions such as Alzheimer's disease, dementia and Parkinson's disease that could also apply to mental health care. The strategies included linking with community partners, employing community health workers, recruiting personnel from the community, and caregiver support. A related study is that of (69) who analyzed the role of context and self-determination in promoting overall health of people with intellectual disabilities. The review highlighted the importance of self-determination and the role thereupon of education and financial autonomy.

A review examined what is known about the impact of integrated care programs on improving mental health equity, with special emphasis on collaborative care (68). This review provides evidence that collaborative care is a model that has the potential to reduce disparities for ethnic minorities and other at-risk populations who are often poorly served by usual primary care systems, and who have lower engagement and health outcomes because of other underlying risk factors. As a systems-based approach, collaborative care has been shown to also improve the quality of care received and health outcomes. The collaborative care team is led by a primary care provider, and includes behavioral health care managers, psychiatrists, and frequently other mental health professionals. The team implements measurement-guided care plans based on evidence-based practice guidelines and focuses particular attention on patients not meeting their clinical goals.

The reviews on healthcare access barriers and improvement strategies were general (70) or they targeted persons with disabilities (71), indigenous people (72), or migrants and refugees (in New-Zealand) (73). A review (70) carried out an environmental scan using an international brief survey to identify innovations in this area according to researchers, practitioners, policymakers and other stakeholders. Over 200 unique innovations to help people living in vulnerable situations to access healthcare were identified. Most innovations addressed supply-side dimensions of access, such as appropriateness and approachability (according to Levesque's framework (74), with less focus on demand-side dimensions. Most innovations were funded by governments and were implemented in the realm of community health. Davy et al. (72), in their study including high-income countries, South America and Papua New Guinea, identified numerous barriers that indigenous peoples face in accessing primary health care, including discrimination, high healthcare costs, and broader social determinants of health such as unemployment. The authors highlighted the need for tailored healthcare services, for employing staff from local indigenous communities, for providing transport facilities, and for reducing healthcare costs for low-income individuals. A review on the impact of various reimbursement systems on health equity (75) found that the impact depended on the specific outcomes studied, the context, and social factors beyond race or socio-economic status. Designing reimbursement systems that address the greater healthcare needs of underserved populations was recommended. The other reviews on healthcare access (71, 73) focused primarily on barriers—possibly because improvement initiatives are not well-documented. Barriers to access by people with disabilities or by immigrants/refugees are considered structural or related to health systems, they are attitudinal, or else they are attributable to social determinants. Among the structural or social barriers, healthcare and specialized equipment costs, language issues (for immigrants and refugees) and logistics constraints are present. Attitudinal barriers include discrimination, negative attitudes, or lack of cultural competence among health care providers. Other health system-related barriers pertain to leadership and policies, as well as the paucity of disaggregated information.

The role of the Global Alliance for Vaccines and Immunization (GAVI) initiatives in reducing inequities in vaccine access and immunization has been widely praised. A review showed that GAVI had significantly improved immunization coverage in eligible countries through targeted policies and supply strategies (76). The paper highlighted, however, that approaches such as tiered pricing have created inequities between GAVI-eligible and ineligible middle-income countries. This was before the COVID-19 pandemic which illustrated inequalities in access to prevention and treatment in a dramatic way. Strategies and proposals to address inequities in testing, vaccines, and treatment for COVID-19 as reviewed by Bell et al. (77) included mobile testing and vaccination centers; at the global level, COVAX and the Fair Priority model; linguistically and culturally tailored medical care for COVID-19; telemedicine to limit face-to-face interactions; and addressing structural causes of inequities such as racism through whole-of society approaches. However, the real impact of these measures is not documented. Other groups exposed to health inequities are those with marginalized social identities because of race, gender and sexual orientation. In a commentary informed by a literature review on social interventions and inputs from health equity-involved stakeholders, Peek et al. (78) listed several recommendations for the Agency for Healthcare Research and Quality to advance health equity in such groups: research on SDOH and the effectiveness and implementation of social care interventions in the healthcare sector; working with other federal agencies to develop complementary approaches addressing social marginalization; and requiring social care intervention researchers to use methods that advance our understanding of social health equity.

## 4 Discussion

This scoping review of reviews on actions to improve health equity was undertaken to describe the types of actions, to identify knowledge gaps, and to recommend approaches integrating health equity and One Health initiatives. The authors had initially intended to document the links between, and interconnections of, health equity and One Health initiatives, and to make recommendations to integrate the two. However, we did not identify any review linking health equity with human-animal-ecosystem health. Only two reviews addressed climate change or action on health inequities (37, 45), and none integrated animal and human health equity. Among the negative impacts of the ongoing climate change and its increasing acceleration, there are changes in food and water supply which hamper nutritional security. Climate and environmental changes induce disproportionate adverse effects on specific populations, which are associated with risks of exacerbating, among others, existing gender and socio-economic inequities. It also affects populations' health outcomes, either directly or indirectly. These range from populations' physical and mental health and wellbeing to the emergence and distribution of vector-borne disease patterns. As only reviews are included in the present paper, however, relevant intervention studies may have been missed. Notwithstanding, this gap underlines the need for health equity concerns to be integrated into One Health endeavors.

The One Health approach and its implementation may be challenging and even more so if one attempts to integrate health equity. Four United Nations technical agencies have developed a joint One Health plan of action for the period 2022–2026 (79). The need for equity is stated but not the strategies. We designed the following cross-table (Table 2) to provide broad examples of interventions addressing various dimensions of One Health and health equity (using the WHO building blocks). Evidently, this table is not based on our review which only captured initiatives in human health, that is, the first row of the table, where more specific examples based on the review are added. In this table, we use “priority populations” instead of marginalized, vulnerable, underserved or minoritized groups, as the groups most in need and therefore targeted will depend on the context and the type of action. Despite the lack of review data connecting health equity with animal and ecosystem health, such a matrix is deemed relevant as a tool to better articulate equity and One Health endeavors. It is noted that whether for animal or ecosystem health, much like for human health, advancing equity means that priority populations are targeted. For example, in the area of service delivery, improving veterinary services or monitoring of drinking water quality would first consider the specific needs of locally neglected, marginalized or socially disadvantaged groups.

**Table 2:** Health equity actions for One Health.

Health equity target	Governance and policy	Information and evidence data	Technologies	Human resources	Service delivery
Human health	Universal health insurance *Institutional capacity for health equity assessment and Integration into policy*	Tailoring health literacy initiatives with and for priority populations *Disaggregated data according to social determinants of health*	Community spaces and services with and for priority populations *Digital health for priority populations*	Inclusive and diversified health workforce *Health equity training for health personnel*	Tailored screening programs for increased access and effective use by priority populations *Community-based tailored prevention programs*
Animal health	Regulation for Standards of Practice for animal health protection with and for priority populations	Tailored agriculture and husbandry literacy with and for priority populations	Clean and safe drinking and irrigation water with and for priority populations	Inclusive and diversified husbandry and harvesting strategies	Tailored community-based animal health services with and for priority populations
Ecosystem health	Regulation of heavy polluting industries proximal to population centers and related damaged green space	Inclusive and diversified environmental outreach initiatives with and for priority populations	Tailored measures to adapt to adverse climate events with and for priority populations	inclusive and diversified strategies to address the intersecting determinants of an ecosystem	Tailored community-based monitoring systems for water quality with and for priority populations

The human health equity actions in italics are practical examples provided in the reviews analyzed.

Using the WHO building blocks for this matrix, as well as to categorize the reviews that were analyzed, helps to better understand key areas for future action and research. For instance, “human resources” was the area with fewer reviews although the critical role of human resources for the delivery of comprehensive healthcare with a focus on social justice was a strong theme across the reviews. Yet institutional capacity to assess health equity or to integrate health equity into practice guidelines was central to some reviews. Gaps were identified in support of a diverse healthcare workforce, especially in relation to Indigenous communities. Training the next generation of healthcare providers at various levels of the health system is essential for embedding equity principles within the One Health framework.

Only a few reviews reported on the impact of interventions on health equity (26, 27, 34, 37, 57, 72) although many emphasized the importance of addressing SODH for reducing disparities, notably in managing chronic conditions. We also identified gaps in how healthcare systems currently assess equity, underscoring the need for more robust tools for measuring and promoting health equity. Braveman's overlapping core principles (11) appear promising for addressing health inequities, particularly when applied alongside the One Health approach: (1) Social justice; (2) Removing obstacles to health for disenfranchised, marginalized, and excluded groups; and (3) Addressing the determinants of health, not only healthcare.

**Figure 2:**
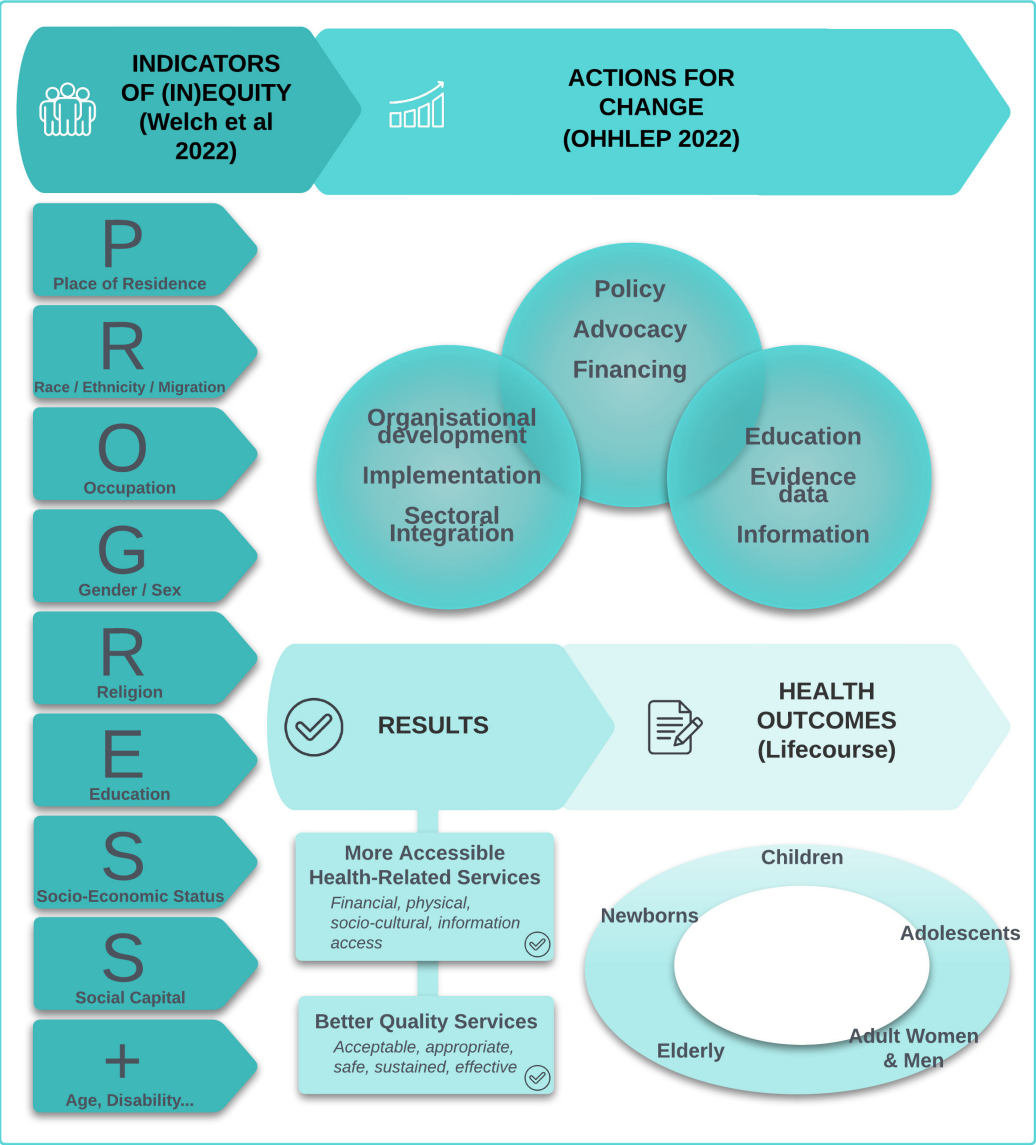
Conceptual framework—improving health equity.

We proposed a conceptual framework to assess the impact of One Health initiatives on equity (Figure 2). It includes on the left the main indicators of inequity of PROGRESS+ (11), or social determinants of health, which can help to target those most in need: Place of residence; Race, ethnicity, migration status; Occupation; Gender and sex; Religion; Education; Socio-economic status; Social capital; and Age, disability, etc. To the right of these determinants, the three broad types of actions for change according to the One Health High Level Panel are shown, that is, policy, implementation and education. The five WHO building blocks describing the One Health actions in Table 1 could have been used instead to categorize the actions: Governance and policy; Information and evidence; Technologies; Human resources; and Service delivery. The expected results in terms of better health equity are better access to health-related services and better quality of such services. Another dimension of equity is in health outcomes throughout the lifecourse. Specific results and outcome indicators would be defined according to the type of One Health actions, as summarized in Table 2; These indicators are to be measured in the targeted priority population as defined based on PROGRESS+. This framework and its parameters will soon be validated as tools for the design and evaluation of equity-sensitive One Health interventions.

Social determinants but also geopolitical and planetary determinants of health inequities must be increasingly considered (80). There is a need for further exploration of how economic policies, education, and sociocultural contexts shape access to health-related services. Multilevel interventions that address structural determinants are crucial for advancing equity.

“Diversity science” was introduced as a framework that, alongside “proportionate universalism,” can help bridge the gap between interventions aimed at the general population and those designed for socially disadvantaged groups (81). Proportionate universalism, which promotes population-wide interventions with additional focus on marginalized communities, is recognized as key to ensuring equitable healthcare access under the One Health framework (82).

The increasing role of digital literacy as a new determinant of health, especially in access to healthcare services, was highlighted in several reviews. Similarly, the role of artificial intelligence (AI) in exacerbating or mitigating inequities was reviewed in at least one paper (48). AI and its impact on health equity are particularly important in agricultural sectors, where One Health interventions are particularly relevant. Reviews demonstrated that a holistic, system-level approach, combined with institutional-level interventions, is necessary to achieve health equity and reduce fragmentation of actions under One Health.

The papers emphasized the importance of addressing power imbalances, engaging with communities, and tackling other root causes of health inequities. Local-level approaches were shown to have the potential for systemic impact, particularly in chronic disease management and prevention. However, long-term outcomes of community engagement and local interventions were not thoroughly explored. The role of participatory implementation science in this regard was emphasized (33). Social needs interventions and neighborhood-scale initiatives—often situated outside formal health systems—can play a critical role in advancing health equity. These strategies resonate with One Health principles by recognizing the interconnectedness of human, animal, and environmental health and the importance of localized responses.

The importance of data is stressed in many reviews. Evidence clearinghouses were identified as essential tools for promoting health equity, although challenges remain in ensuring their methods are transparent and equity focused. Disaggregated and context-sensitive health data for identifying and addressing barriers according to specific intersecting demographic factors and social determinants of health such as age, socio-economic and immigration status, sex, gender, and disability are essential, as shown in many of the reviews included in this paper. Disaggregating health data to reveal disparities and tailor policies was seen as vital, though operational barriers, such as data privacy concerns and technological limitations, hinder widespread implementation. Systematic disaggregation of health data is critical for creating evidence-based policies that directly address inequities, aligning with One Health's integrated, cross-sectoral approach.

The authors also noted that the number of health equity-focused reviews has increased over the last few years, indicating a growing concern about health inequities, which may partly result from the COVID-19 pandemic, even if only one review dealt with inequities in the prevention and treatment of COVID-19 (77). Most papers were from high-income countries, particularly the USA, where yet little progress in health equity is observed (83). Only a few reviews included LMICs (20, 38, 51, 64, 73), where health inequities are likely even more marked and where more equity-focused action is called for.

As pointed out in the introduction of this article, all the papers that were included in this scoping review were only focusing on humans, especially certain segments of the human population. Considering health equity without understanding the interconnectedness and interdependence of the human population with nature—animals, insects, soil, air, and water. The authors point out that without this understanding, it is of limited value to discuss health equity.

At variance with systematic and even narrative reviews, scoping reviews do not generally include an assessment of the quality of the selected studies, and this is the case for the present review. On the one hand, we did not think it was possible to assess the quality of the reviews we analyzed unless we excluded the overviews which usually do not follow a strict methodology. We felt that excluding the latter would have meant losing much relevant information since it was the case for roughly one fifth of the selected papers. On the other hand, it would have been difficult to appraise the quality of the “standard” reviews as these included systematic, narrative, integrative and scoping reviews, as well as a few umbrella reviews, with different quality criteria. According to Grant and Booth (84), the absence of review quality information may limit the uptake of the findings into policy and practice. However, the consultation with stakeholders, even if it is planned only after the publication of the review, may confer additional meaning and applicability to the scoping study, and indeed this consultation is considered by Levac et al. (15) as the last stage of the process and as a knowledge transfer mechanism.

Another limitation is that we did not report the number of hits per database or the number of duplicates as this is not required for reviews of reviews (15). Limitations associated with an approach such as review of reviews could be addressed in future work by prospectively recording database-specific yields and duplicate records using reference management software or systematic review management platforms, thereby enhancing transparency and reproducibility.

## 5 Conclusion

This review of reviews on actions to improve health equity highlights the need for clearer, more nuanced definitions of health equity within One Health contexts and beyond. Without a clearer understanding and empirical evidence, it remains challenging to establish good practices for integrating health equity into One Health interventions. While diversity science and proportionate universalism offer promising frameworks, their practical impact on health equity requires further investigation into the realities of diverse epistemologies and “sciences” from various parts of the world, especially in low- and middle- income countries (LMICs), where One Health and equity challenges are most acute. A priority research need is to evaluate One Health programs by their equity impact and not only their effectiveness, using selected equity variables as indicators. Examples of such variables are access to healthcare, veterinary services, clean water or green spaces, exposure to environmental contaminants, traditional ecological knowledge. The review also underscores the importance of learning from local communities by collaborating with them as equal partners, supporting a diverse health workforce, and improving data-driven (not only quantitative, but also qualitative) policy solutions to advance equity. Moreover, addressing the challenges and opportunities posed by digital health and AI, and particularly their role as potential effect modifiers in health inequities, should be prioritized in future research. By systematically integrating a health equity lens (principles and indicators) within One Health approaches, interventions can more effectively reduce disparities and help achieve the SDGs. Adopting a health equity lens means always considering priority populations, that is, the marginalized or socially disadvantaged groups, and their needs when designing and implementing One Health initiatives.

## Footnotes

1 Working groups focus on: Environmental health; Sustainable food systems; One health and urban settings; Equitable access to health-related services; Financing for sustainable health; Governance and policy; Education and communication; One health next generation; and One health in humanitarian settings.

## Appendix

**Table A1:** Concept plan for literature search.

Health inequities Health disparities Healthcare disparities Health vulnerability	Access to services Accessibility of services Physical access Financial access Access to information Quality of services Effectiveness Sustainability Safety Digital connectivity Empowerment Resilience Environmental sustainability	Health equity Universal health coverage Inclusive health Health equality Health justice Right to health One health Sustainable
